# Total Synthesis of the Proposed Structure of Paraphaeosphaeride C †

**DOI:** 10.3390/molecules24234230

**Published:** 2019-11-21

**Authors:** Kenichi Kobayashi, Risako Kunimura, Hiroshi Kogen

**Affiliations:** Graduate School of Pharmaceutical Sciences, Meiji Pharmaceutical University, Tokyo 204-8588, Japan; r.kunimura@hotmail.com

**Keywords:** paraphaeosphaeride C, STAT3, anti-cancer, total synthesis, stereoselective synthesis

## Abstract

Paraphaeosphaeride C is a demethoxy derivative of phaeosphaeride A and exhibits STAT3 inhibitory activity. Our previous papers reported the total synthesis of phaeosphaeride A using a diastereoselective vinyl anion aldol reaction as the key step to construct the dihydropyran ring. In this work, the first total synthesis of the proposed structure of paraphaeosphaeride C was achieved via a similar synthetic strategy. The synthetic compound was characterized through extensive nuclear magnetic resonance (NMR) analysis but the ^1^H and ^13^C-NMR data for this compound did not correspond to those reported in the literature for paraphaeosphaeride C.

## 1. Introduction

**Figure 1:**
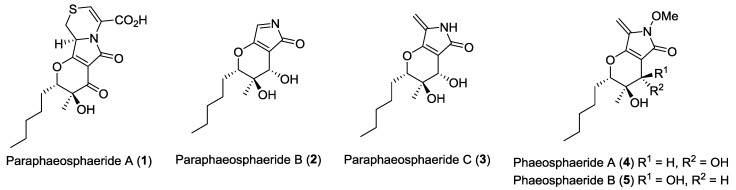
Structures of paraphaeosphaerides A–C (**1**–**3**) and phaeosphaerides A (**4**) and B (**5**).

Paraphaeosphaerides A–C (**1**–**3**, Figure 1) were isolated from *Paraphaeosphaeria neglecta* FT462 by Cao and co-workers in 2015 [1] and these natural products are structurally related to phaeosphaerides A (**4**) and B (**5**) [2]. Among them, paraphaeosphaeride C (PPPC, **3**), which is a demethoxy derivative of phaeosphaeride A (**4**), was reported to exhibit STAT3 inhibitory activity at 10 μM against MDA-MB-231 breast cancer cells. In addition, **3** was reported to be active against A2780 and A2780cisR (cisplatin-resistant A2780) cells with IC_50_ values of 15.0 and 52.4 μM, respectively. Thus, **3** might be a potential lead compound for anti-cancer drug discovery alongside phaeosphaeride A [3,4,5,6,7,8].

**Scheme 1:**
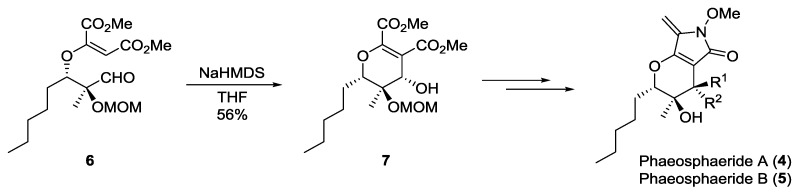
Our synthesis of phaeosphaerides A (**4**) and B (**5**) featuring a vinyl anion aldol reaction.

In our previous work, we achieved the total synthesis of phaeosphaerides A (**4**) and B (**5**), using a diastereoselective vinyl anion aldol reaction of aldehyde **6** to form **7** as the key step for assembling the dihydropyran core (Scheme 1) [9,10,11].

We envisaged that a similar synthetic strategy could be applied to the synthesis of paraphaeosphaeride C (**3**) from the same intermediate **7**. This paper describes the first total synthesis of the proposed structure of paraphaeosphaeride C (**3**).

## 2. Results and Discussion

**Scheme 2:**
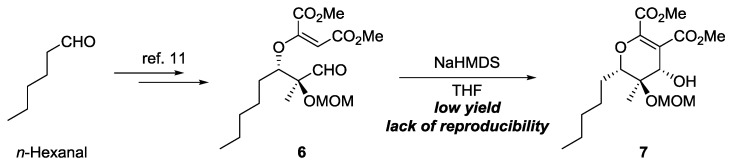
Unsuccessful formation of dihydropyran derivative **7** from aldehyde **6**. (Reproduced with permission from [11].)

Our synthesis commenced with the preparation of the key synthetic intermediate **7** used to obtain phaeosphaerides A and B (Scheme 2). According to our previously reported protocol [11], *n*-hexanal was converted over 10 steps into aldehyde **6**, which was then subjected to the vinyl anion aldol reaction using sodium bis(trimethylsilyl)amide (NaHMDS) in THF. In our previous work, dihydropyran derivative **7** was obtained in 56% yield but the reaction unexpectedly suffered from low yield and lack of reproducibility. Several batches of NaHMDS and THF from different lots and suppliers were screened; nevertheless, the yield of product **7** did not improve. Thus, re-examination of this reaction was required.

**Figure 2:**
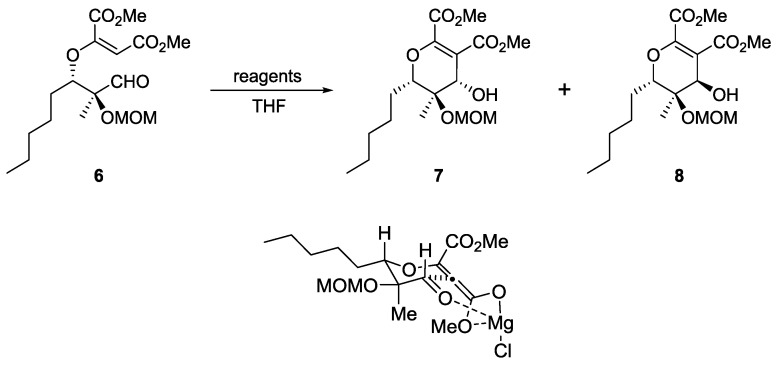
Plausible Mg^2+^-stabilized transition state leading to **7**.

We initially attempted to use lithium tetramethylpiperidide (LiTMP) prepared from tetramethylpiperidine (TMP) and *n*-BuLi in situ (Table 1). When TMP (1.5 equiv) and *n*-BuLi (1.5 equiv) were used, the desired product **7** and its diastereomer **8** were obtained in 21% and 8% yield, respectively, along with recovery of the starting aldehyde **6** in 40% yield (entry 1). Upon doubling the amount of each reagent, the yield of **7** improved to 42% but remained unsatisfactory (entry 2). Further increasing the amounts of TMP and *n*-BuLi apparently diminished the combined yield of the products, albeit with no recovery of aldehyde **6** (entry 3). To our delight, use of the Knochel-Hauser base TMPMgCl·LiCl (3 equiv) [12,13,14] afforded **7** in 82% yield and significantly improved stereoselectivity (entry 4). Furthermore, this reagent reproducibly delivered the desired product **7** in high yield. In this reaction, we suppose that the strong basicity of TMPMgCl·LiCl is crucial and the divalent magnesium ion might stabilize the transition state leading to **7** (Figure 2), because monovalent metal cations (Li, Na and K) resulted in low yield and poor stereoselectivity in our initial experiments [9].

**Scheme 3:**
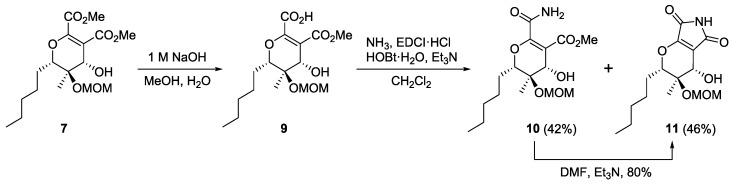
Synthesis of bicyclic compound **11**.

**Scheme 4:**
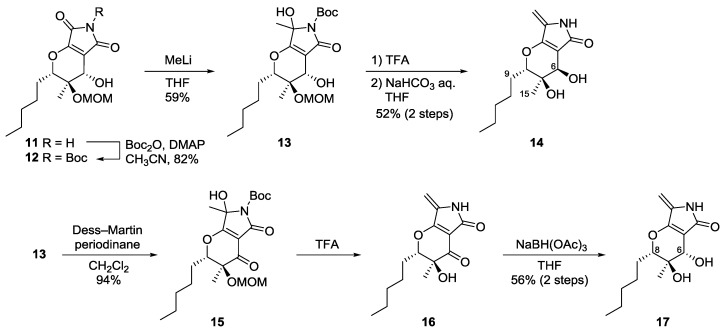
Total synthesis of the proposed structure of paraphaeosphaeride C.

With suitable reaction conditions for the vinyl anion aldol reaction in hand, our attention next turned to further functional group transformations toward the synthesis of PPPC (**3**) (Scheme 3 and Scheme 4). Following our previously reported method [9,10,11], diester **7** was regioselectively hydrolyzed using aqueous NaOH to afford monoacid **9**, which was immediately reacted with ammonia to form amide **10** along with imide **11** in 42% and 46% yield, respectively. Upon heating **10** with Et_3_N in DMF at 70 °C, imide **11** was efficiently obtained in 80% yield (Scheme 3).

Having synthesized bicyclic compound **11**, the remaining task for the total synthesis was achieving regioselective *exo*-methylenation. Thus, **11** was treated with MeMgBr according to the literature [9,10,11], although only unreacted **11** was recovered. The use of more reactive MeLi also resulted in no reaction. Then, the imide nitrogen in **11** was initially protected with a Boc group to obtain **12** in 82% yield. Subsequent reaction of **12** with MeLi in THF proceeded successfully to furnish hemiaminal **13** in moderate yield (59%), although MeMgBr remained ineffective (Scheme 4).

For dehydration of the hemiaminal moiety and removal of both the Boc and MOM protecting groups to directly convert **13** to PPPC, treatment of **13** with an acid (HCl/1,4-dioxane [9,10,11] or *p*-TsOH/toluene [3,4]) resulted in complex mixtures of products. However, upon using trifluoroacetic acid (TFA), dehydration and deprotection occurred smoothly to produce a trifluoroacetylated product, which was hydrolyzed with aqueous NaHCO_3_ to afford diol **14** in 52% yield from **13**, the nuclear magnetic resonance (NMR) spectra of which were not consistent with the literature data for PPPC. Diol **14** may be the C-6 epimer of PPPC formed via stereoinversion at C-6, as observed in the synthesis of phaeosphaeride B (**5**) [11]. The configuration at C-6 in **14** was established using NOESY correlations between H-6 and H-9 and between H-6 and H-15.

To avoid similar stereoinversion at C-6, alcohol **13** was protected via oxidation with Dess-Martin periodinane to afford ketone **15** (94%), which underwent the expected conversion to alcohol **16** upon treatment with TFA. Owing to its instability, **16** was used in the next step without purification. Finally, stereoselective reduction of **16** was accomplished using NaBH(OAc)_3_ to afford the desired diol **17** in 56% yield from **15**, whereas attempted reduction with LiAlH_4_, DIBAL-H or NaBH_4_/CeCl_3_ provided complex mixtures of products.

**Figure 3:**
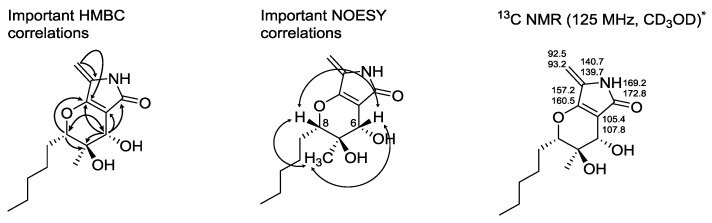
Structural characterization of synthetic compound **17** (* Upper values: PPPC (**3**) [1], Lower values: Synthetic **17**; Reproduced with permission from [1]).

The gross structure of synthetic **17** was deduced to be the proposed structure PPPC (**3**) via extensive NMR studies including HMBC and NOESY experiments, as summarized in Figure 3. NOESY correlations suggested a *syn* relationship between H-6 and H-8, with a flexible conformation of the dihydropyran ring. However, the ^1^H and ^13^C-NMR spectra of **17** did not match those reported in the literature for PPPC [1]. Upon comparing the ^13^C-NMR spectra of **3** and **17**, slight differences were observed in the upper five-membered ring, whereas the signals originating from the lower dihydropyran ring were in almost complete agreement (see Supplementary Materials). In addition, the optical rotation of synthetic **17** (${\left[\alpha \right]}_{D}^{25}$ + 38.6 (*c* 0.08, MeOH)) was not identical to that of the reported natural product **3** (${\left[\alpha \right]}_{D}^{25}$ − 128.6 (*c* 0.14, MeOH)). These results suggest that PPPC may have a five-membered ring different from that reported in the literature connected to the dihydropyran moiety, although the true structure of PPPC remains uncertain.

In conclusion, we have achieved the first total synthesis of the proposed structure of paraphaeosphaeride C. This synthesis featured a TMPMgCl·LiCl-mediated vinyl anion aldol reaction and subsequent appropriate functional group transformations, including regioselective methylenation and stereoselective reduction to establish the C-6 stereocenter. In addition, this work implies that the structure of natural paraphaeosphaeride C was incorrectly assigned. Further studies toward the structural assignment of the natural product are currently underway in our group.

## 3. Materials and Methods

### 3.1. General Experimental Methods

^1^H-NMR spectra were recorded on a JEOL JNM-AL300 (300 MHz), JEOL JNM-ECS400 (400 MHz) or JEOL JNM-ECA500 (500 MHz) (JEOL Ltd., Akishima, Japan) instrument. The chemical shifts are expressed in ppm relative to tetramethylsilane (δ = 0) as an internal standard (CDCl_3_ or CD_3_OD solution). Splitting patterns are indicated as follows—s, singlet; d, doublet; t, triplet; m, multiplet; br, broad peak. ^13^C-NMR spectra were recorded on a JEOL JNM-ECA500 (125 MHz) or JEOL JNM-ECS400 (100 MHz) instrument. The chemical shifts are reported in ppm relative to the central line of the triplet at 77.0 ppm for CDCl_3_ or that of the septet at 49.3 ppm for CD_3_OD. Infrared (IR) spectra were measured on a JASCO VALOR-III spectrometer (JASCO Corporation, Hachioji, Japan) and are reported in wavenumbers (cm^−1^). Both low-resolution mass spectra (MS) and high-resolution mass spectra (HRMS) were obtained using a JEOL JMS 700 (FAB mode) instrument with a direct inlet system. Optical rotations were measured on a JASCO P-2200 polarimeter using a cell with an optical path length of 100 mm. Column chromatography was performed on silica gel (40–100 mesh, FUJI SILYSIA CHEMICAL Ltd., Kasugai, Japan). Analytical thin-layer chromatography (TLC) was conducted using 0.25 mm silica gel 60 F plates.

### 3.2. Experimental Procedures and Characterization Data

#### 3.2.1. Dimethyl (*2S*,*3R*,*4S*)-4-Hydroxy-3-Methoxymethoxy-3-Methyl-2-Pentyl-3,4-Dihydro-*2H*-Pyran-5,6-Dicarboxylate (**7**)

To a stirred solution of 2,2,6,6-tetramethylpiperidinylmagnesium chloride-lithium chloride complex (1.0 M solution in THF/toluene, 2.3 mL, 2.3 mmol) in THF (8 mL) was added a solution of 6 (269 mg, 0.746 mmol) in THF (6 mL) at −78 °C and the mixture was stirred at the same temperature for 20 min. Saturated aqueous NH_4_Cl and H_2_O were then added to the reaction mixture. The resulting mixture was extracted with Et_2_O and the combined organic layers were dried over Na_2_SO_4_ and concentrated in vacuo. The crude product was purified by column chromatography on silica gel (*n*-hexane/EtOAc 3:1→7:3) to afford 7 (219 mg, 82%) and 8 (26.1 mg, 9%) as colorless oils. The spectral data for 7 and 8 were identical to those reported in the literature [11].

#### 3.2.2. Methyl (*2S*,*3R*,*4S*)-6-Carbamoyl-4-Hydroxy-3-Methoxymethoxy-3-Methyl-2-Pentyl-3,4-Dihydro-*2H*-Pyran-5-Carboxylate (**10**) and (*2S*,*3R*,*4S*)-4-Hydroxy-3-Methoxymethoxy-3-Methyl-2-Pentyl-3,4-Dihydropyrano[2,3-c]pyrrole-5,7(*2H*,*6H*)-Dione (**11**)

To a stirred solution of **7** (169 mg, 0.469 mmol) in MeOH (6 mL) was added 1 M aqueous NaOH (0.9 mL, 0.9 mmol) at room temperature. The resulting mixture was stirred at 35 °C for 15 h and then concentrated in vacuo. The residual oil was dissolved in H_2_O and 1 M aqueous HCl was added to this solution. The mixture was extracted with EtOAc and the combined organic layers were dried over Na_2_SO_4_ and concentrated in vacuo. The crude monoacid **9** was used in the next step without further purification. The crude product was dissolved in CH_2_Cl_2_ (16 mL) and NH_3_ (7 N solution in MeOH, 0.10 mL, 0.70 mmol), Et_3_N (0.15 mL, 1.1 mmol), HOBt∙H_2_O (90 mg, 0.59 mmol) and EDCI∙HCl (107 mg, 0.56 mmol) were added to this solution at room temperature. The mixture was stirred for 18 h. After addition of H_2_O, the layers were separated and the aqueous layer was extracted with CH_2_Cl_2_. The combined organic layers were dried over Na_2_SO_4_ and concentrated in vacuo. The crude product was purified by column chromatography on silica gel (*n*-hexane/EtOAc 3:2→1:1→3:7→1:4) to afford **10** (67.3 mg, 42%) and **11** (67.1 mg, 46%) as colorless oils.

Conversion of **10** to **11**—A solution of **10** (58.7 mg, 0.17 mmol) and Et_3_N (0.24 mL, 1.7 mmol) in DMF (1.7 mL) was stirred at 70 °C for 7 h. After concentration, the crude product was purified by column chromatography on silica gel (*n*-hexane/EtOAc 3:2) to afford **11** (42.4 mg, 80%) as a colorless oil.

Spectral data for **10**: ${\left[\alpha \right]}_{D}^{24}$ + 215.4 (*c* 0.23, CHCl_3_); IR (neat/NaCl) 3335, 2958, 1686, 1654, 1436, 1261, 1029, 799 cm^−1^; ^1^H-NMR (300 MHz, CDCl_3_) δ 6.25 (br s, 1H), 6.05 (br s, 1H), 4.97 (d, *J* = 7.6 Hz, 1H), 4.64 (d, *J* = 8.0 Hz, 1H), 4.59 (d, *J* = 3.2 Hz, 1H), 4.20 (d, *J* = 4.0 Hz, 1H), 3.87 (dd, *J* = 1.6, 10.0 Hz, 1H), 3.81 (s, 3H), 3.43 (s, 3H), 1.90–1.77 (m, 2H), 1.65–1.47 (m, 2H), 1.42–1.25 (m, 5H), 1.22 (s, 3H), 0.91 (t, *J* = 6.8 Hz, 3H); ^13^C-NMR (100 MHz, CDCl_3_) δ 167.4, 162.7, 144.3, 114.9, 91.1, 81.7, 75.7, 72.1, 56.0, 52.5, 31.6, 27.2, 26.1, 22.5, 14.0, 9.8; MS (FAB+) *m*/*z*: 346 [M + H]^+^; HRMS (FAB+) *m*/*z*: [M + H]^+^ Calcd for C_16_H_28_NO_7_ 346.1866; Found 346.1864.

Spectral data for **11**: ${\left[\alpha \right]}_{D}^{20}$ − 60.3 (*c* 0.067, CHCl_3_); IR (neat/NaCl) 3450, 3266, 2926, 1730, 1672, 1031, 801 cm^−1^; ^1^H-NMR (400 MHz, CDCl_3_) δ 6.93 (br s, 1H), 4.97 (d, *J* = 8.0 Hz, 1H), 4.67 (d, *J* = 8.0 Hz, 1H), 4,63 (br s, 1H), 4,11 (br d, *J* = 8.0 Hz, 1H), 3.45 (s, 3H), 1.87–1.82 (m, 1H), 1.77–1.65 (m, 2H), 1.43–1.28 (m, 6H), 1.25 (s, 3H), 0.90 (t, *J* = 7.0 Hz, 3H); ^13^C-NMR (125 MHz, CDCl_3_) δ 167.4, 163.4, 155.4, 112.4, 91.2, 85.9, 77.2, 69.0, 56.2, 31.6, 27.7, 26.0, 22.5, 14.0, 10.3; MS (FAB+) *m*/*z*: 314 [M + H]^+^; HRMS (FAB+) *m*/*z*: [M + H]^+^ Calcd for C_15_H_24_NO_6_ 314.1604; Found 314.1611.

#### 3.2.3. Tert-Butyl (*2S*,*3R*,*4S*)-4-Hydroxy-3-Methoxymethoxy-3-Methyl-5,7-Dioxo-2-Pentyl-3,4,5,7-Tetrahydropyrano[2,3-c]pyrrole-6(*2H*)-Carboxylate (**12**)

To a stirred solution of **11** (48.2 mg, 0.154 mmol) in CH_3_CN (2.5 mL) were added DMAP (1.8 mg, 0.015 mmol) and di-*tert*-butyl dicarbonate (42.0 mg, 0.192 mmol) at room temperature. After stirring for 2 h, the reaction mixture was concentrated in vacuo. The crude product was purified by column chromatography on silica gel (*n*-hexane/EtOAc 3:1) to afford **12** (50.6 mg, 82%) as a colorless oil.

Spectral data for **12**: ${\left[\alpha \right]}_{D}^{24}$ − 84.2 (*c* 0.10, CHCl_3_); IR (neat/NaCl) 3516, 2958, 1769, 1680, 1258, 1094, 1027, 801 cm^−1^; ^1^H-NMR (400 MHz, CDCl_3_) δ 4.92 (d, *J* = 7.5 Hz, 1H), 4.70 (d, *J* = 7.5 Hz, 1H), 4.59 (br s, 1H), 4.25–4.05 (m, 1H), 3.42 (s, 3H), 1.82–1.72 (m, 1H), 1.69–1.58 (m, 1H), 1.57 (s, 9H), 1.41–1.21 (m, 7H), 1.28 (s, 3H), 0.90 (t, *J* = 7.0 Hz, 3H); ^13^C-NMR (125 MHz, CDCl_3_) δ 166.2, 163.8, 154.7, 145.9, 112.8, 91.3, 86.2, 85.0, 77.2, 76.8, 56.1, 31.5, 27.9 (3C), 27.7, 26.1, 22.5, 14.0, 10.1; MS (FAB+) *m*/*z*: 414 [M + H]^+^; HRMS (FAB+) *m*/*z*: [M + H]^+^ Calcd for C_20_H_32_NO_8_ 414.2128; Found 414.2134.

#### 3.2.4. Tert-Butyl (*2S*,*3R*,*4S*)-4,7-Dihydroxy-3-Methoxymethoxy-3,7-Dimethyl-5-Oxo-2-Pentyl-3,4,5,7-Tetrahydropyrano[2,3-c]pyrrole-6(*2H*)-Carboxylate (**13**)

To a stirred solution of **12** (8.7 mg, 0.021 mmol) in THF (2 mL) was added MeLi (1.16 mmol/mL solution in Et_2_O, 0.050 mL, 0.06 mmol) at −78 °C. The resulting mixture was stirred at the same temperature for 20 min. The reaction was quenched with saturated aqueous NaHCO_3_ and the mixture was extracted with CH_2_Cl_2_. The combined organic layers were dried over Na_2_SO_4_ and concentrated in vacuo. The crude product was purified by column chromatography on silica gel (*n*-hexane/EtOAc 3:2) to afford **13** (5.1 mg, 59%, 1:1 inseparable mixture) as a colorless oil.

IR (neat/NaCl) 3412, 2927, 1759, 1688, 1330, 1151, 1033, 752 cm^−1^; ^1^H-NMR (500 MHz, CDCl_3_) δ 4.87 (d, *J* = 7.5 Hz, 1H × 1/2), 4.86 (d, *J* = 7.5 Hz, 1H × 1/2), 4.82 (d, *J* = 7.0 Hz, 1H × 1/2), 4.79 (d, *J* = 7.5 Hz, 1H × 1/2), 4.56 (br s, 1H × 1/2), 4.52 (br s, 1H × 1/2), 4.43 (br s, 1H × 1/2), 4.40 (s, 1H × 1/2), 4.22–4.08 (m, 1H × 1/2), 4.18–4.00 (m, 1H × 1/2), 3.39 (s, 3H), 1.80 (s, 3H), 1.70–1.19 (m, 8H), 1.57 (s, 9H), 1.33 (s, 3H × 1/2), 1.29 (s, 3H × 1/2), 0.90 (t, *J* = 7.0 Hz, 3H); ^13^C-NMR (125 MHz, CDCl_3_) δ 169.9 (both isomers), 150.7 (both isomers), 150.6 (both isomers), 105.0, 104.9, 91.6 (both isomers), 86.1, 85.9, 85.3 (both isomers), 83.8, 83.7, 83.1 (both isomers), 76.5 (both isomers), 56.0, 55.9, 31.62, 31.57, 28.2 (3C, both isomers), 27.94, 27.90, 26.19, 26.17, 23.5, 23.4, 22.55, 22.49, 14.0 (2C, both isomers); MS (FAB+) *m*/*z*: 430 [M + H]^+^; HRMS (FAB+) *m*/*z*: [M + H]^+^ Calcd for C_21_H_36_NO_8_ 430.2441; Found 430.2448.

#### 3.2.5. (*2S*,*3R*,*4R*)-3,4-Dihydroxy-3-Methyl-7-Methylene-2-Pentyl-3,4,6,7-Tetrahydropyrano[2,3-c]pyrrol-5(*2H*)-One (**14**)

Compound **13** (3.3 mg, 0.0077 mmol) was dissolved in TFA (0.3 mL) and the resulting mixture was stirred for 1 h at room temperature. After concentration under vacuum, the crude product was dissolved in THF (0.4 mL). To this solution was added half-saturated aqueous NaHCO_3_ (0.2 mL). The reaction mixture was stirred for 2 h at room temperature and then diluted with saturated aqueous NaCl. The mixture was extracted with Et_2_O and the combined organic layers were dried over Na_2_SO_4_ and concentrated in vacuo. The crude product was purified by column chromatography on silica gel (*n*-hexane/EtOAc 3:7) to afford **14** (1.1 mg, 52%) as a colorless oil.

${\left[\alpha \right]}_{D}^{25}$ + 15.3 (*c* 0.06, MeOH); IR (neat/NaCl) 3303, 3231, 2954, 2927, 2372, 2311, 1698, 1637, 1056, 918 cm^−1^; ^1^H-NMR (500 MHz, CD_3_OD) δ 4.97 (d, *J* = 1.0 Hz, 1H), 4.87 (d, *J* = 1.5 Hz, 1H), 4.04 (dd, *J* = 2.0, 10.5 Hz, 1H), 3.96 (s, 1H), 1.98–1.90 (m, 1H), 1.74–1.58 (m, 2H), 1.52–1.25 (m, 5H), 1.00 (s, 3H), 0.94 (t, *J* = 7.5 Hz, 3H); ^13^C-NMR (125 MHz, CD_3_OD) δ 172.3, 160.3, 139.3, 108.4, 93.8, 82.1, 71.5, 66.2, 32.9, 28.7, 27.4, 23.7, 18.3, 14.4; MS (FAB+) *m*/*z*: 268 [M + H]^+^; HRMS (FAB+) *m*/*z*: [M + H]^+^ Calcd for C_14_H_22_NO_4_ 268.1549; Found 268.1542.

#### 3.2.6. Tert-Butyl (*2S*,*3S*)-7-Hydroxy-3-Methoxymethoxy-3,7-Dimethyl-4,5-Dioxo-2-Pentyl-3,4,5,7-Tetrahydropyrano[2,3-c]pyrrole-6(*2H*)-Carboxylate (**15**)

To a stirred solution of **13** (1.7 mg, 0.0040 mmol) in CH_2_Cl_2_ (0.4 mL) was added Dess-Martin periodinane (3.9 mg, 0.0092 mmol) at room temperature. The reaction mixture was stirred for 5 h and then poured into a mixture of 5% aqueous Na_2_SO_3_, saturated aqueous NaHCO_3_ and H_2_O. The resulting mixture was extracted with Et_2_O and the combined organic layers were dried over Na_2_SO_4_ and concentrated in vacuo. The crude product was purified by column chromatography on silica gel (*n*-hexane/EtOAc 3:2) to afford **15** (1.6 mg, 94%, 6:5 inseparable mixture) as a colorless oil.

IR (neat/NaCl) 3404, 2931, 1768, 1636, 1317, 1151, 1027, 758 cm^−1^; ^1^H-NMR (400 MHz, CDCl_3_) δ 4.85 (dd, *J* = 3.0, 10.0 Hz, 1H × 5/11), 4.81 (dd, *J* = 3.0, 11.0 Hz, 1H × 6/11), 4.78 (d, *J* = 7.5 Hz, 1H), 4.73 (d, *J* = 7.5 Hz, 1H × 5/11), 4.68 (d, *J* = 7.5 Hz, 1H × 6/11), 3.36 (s, 3H × 5/11), 3.35 (s, 3H × 6/11), 1.88 (s, 3H × 6/11), 1.87 (s, 3H × 5/11), 1.80–1.60 (m, 2H), 1.57 (s, 9H), 1.50–1.42 (m, 2H), 1.37–1.30 (m, 4H), 1.35 (s, 3H × 6/11), 1.31 (s, 3H × 5/11), 0.92–0.88 (m, 3H); ^13^C-NMR (125 MHz, CDCl_3_) δ 186.0 (both isomers), 160.1 (both isomers), 150.9 (both isomers), 148.3 (both isomers), 103.1, 103.0, 92.95, 92.89, 90.4, 90.1, 85.9, 85.8, 84.4 (both isomers), 78.4, 78.3, 56.2, 56.1, 31.3 (both isomers), 28.1 (3C, both isomers), 27.52, 27.51, 25.4, 25.2, 23.5, 23.4, 22.4, 22.3, 15.6, 15.5, 13.9 (both isomers); MS (FAB+) *m*/*z*: 428 [M + H]^+^; HRMS (FAB+) *m*/*z*: [M + H]^+^ Calcd for C_21_H_34_NO_8_ 428.2284; Found 428.2289.

#### 3.2.7. (*2S*,*3S*)-3-Hydroxy-3-Methyl-7-Methylene-2-Pentyl-2,3,6,7-Tetrahydropyrano[2,3-c]pyrrole-4,5-Dione (**16**)

Compound **15** (1.7 mg, 0.0040 mmol) was dissolved in TFA (0.15 mL) and the resulting mixture was stirred for 6 h at room temperature. After concentration under vacuum, the crude product was used directly in the next step without further purification.

^1^H-NMR (400 MHz, CDCl_3_) δ 7.40 (br s, 1H), 5.32 (d, *J* = 2.4 Hz, 1H), 5.12 (d, *J* = 2.4 Hz, 1H), 4.36 (dd, *J* = 2.4, 9.6 Hz, 1H), 2.03–1.21 (m, 9H), 1.26 (s, 3H), 0.96–0.88 (m, 3H); ^13^C-NMR (100 MHz, CDCl_3_) δ 190.9, 164.4, 153.8, 135.7, 103.0, 97.7, 89.2, 72.1, 31.4, 27.6, 25.4, 22.5, 18.8, 14.0.

#### 3.2.8. Proposed Structure of Paraphaeosphaeride C (**17**)

To a stirred solution of crude **16** (prepared above) in THF (1.0 mL) was added sodium triacetoxyborohydride (1.3 mg, 0.0061 mmol) at 0 °C. After stirring at the same temperature for 15 min, the mixture was allowed to warm to room temperature and stirring was continued for 1.5 h. As TLC analysis revealed the presence of unreacted **16**, a second portion of sodium triacetoxyborohydride (4.3 mg, 0.020 mmol) was added at 0 °C and the mixture was stirred for an additional 15 min at room temperature. A third portion of sodium triacetoxyborohydride (6.0 mg, 0.028 mmol) was then added at 0 °C and the mixture was stirred for a further 1 h at room temperature. The reaction was then quenched with saturated aqueous NaHCO_3_ and extracted with EtOAc. The combined organic layers were washed with H_2_O and saturated aqueous NaCl, dried over Na_2_SO_4_ and concentrated in vacuo. The crude product was purified by column chromatography on silica gel (EtOAc) to afford **17** (0.6 mg, 56% over two steps) as a colorless oil.

${\left[\alpha \right]}_{D}^{25}$ + 38.6 (*c* 0.08, MeOH); IR (neat/NaCl) 3348, 2954, 2927, 1685, 1638, 1455, 1363, 1262, 1025, 801 cm^−1^; ^1^H-NMR (500 MHz, CD_3_OD) δ 4.94 (d, *J* = 1.0 Hz, 1H), 4.85 (d, *J* = 1.0 Hz, 1H), 4.14 (br s, 1H), 4.10 (br d, *J* = 11.0 Hz, 1H), 1.90 (m, 1H), 1.66 (m, 1H), 1.59 (m, 1H), 1.41 (m, 1H), 1.34 (m, 4H), 1.31 (s, 3H), 0.91 (t, *J* = 7.0 Hz, 3H); ^13^C-NMR (125 MHz, CD_3_OD) δ 172.8, 160.5, 139.7, 107.8, 93.2, 87.8, 72.8, 67.5, 32.6, 29.2, 27.8, 23.6, 19.7, 14.3; MS (FAB+) *m*/*z*: 268 [M + H]^+^; HRMS (FAB+) *m*/*z*: [M + H]^+^ Calcd for C_14_H_22_NO_4_ 268.1549; Found 268.1558.

## Figures and Tables

**Table 1:** Vinyl anion aldol reaction of aldehyde **6**.

Entry	Reagents (equiv)	Temp (°C)	Time (min)	Result ^1^
1	TMP (1.5), *n*-BuLi (1.5)	−40	10	**6** (40%), **7** (21%), **8** (8%)
2	TMP (3.0), *n*-BuLi (3.0)	−40	10	**6** (7%), **7** (42%), **8** (9%)
3	TMP (5.0), *n*-BuLi (5.0)	−40	10	**7** (30%), **8** (5%)
4	TMPMgCl·LiCl (3.0)	−78	20	**7** (82%), **8** (9%)

^1^ Isolated yield.

## Supplementary Materials

Copies of ^1^H and ^13^C-NMR spectra of all new compounds are available online.
