# Supplementary material for: Total Synthesis of the Proposed Structure of Paraphaeosphaeride C
Source: Molecules. 2019 Nov 21;24(23):4230. doi: 10.3390/molecules24234230 (PMC6930451; doi:10.3390/molecules24234230)
Supplement: Supplementary file 1 [file molecules-24-04230-s001.pdf]

Supporting information

Total Synthesis of the Proposed Structure of Paraphaeosphaeride C

Kenichi Kobayashii,* Risako Kunimura, Hiroshi Kogen*

Graduate School of Pharmaceutical Sciences, Meiji Pharmaceutical University
2-522-1 Noshio, Kiyose, Tokyo 204-8588, Japan.

kenichik@my-pharm.ac.jp
hkogen@my-pharm.ac.jp

Table of Contents

^{1}H and ^{13}C NMR Spectra S2-S20

10-1H

C:¥Users¥kogen¥Desktop¥data¥10-1H.als

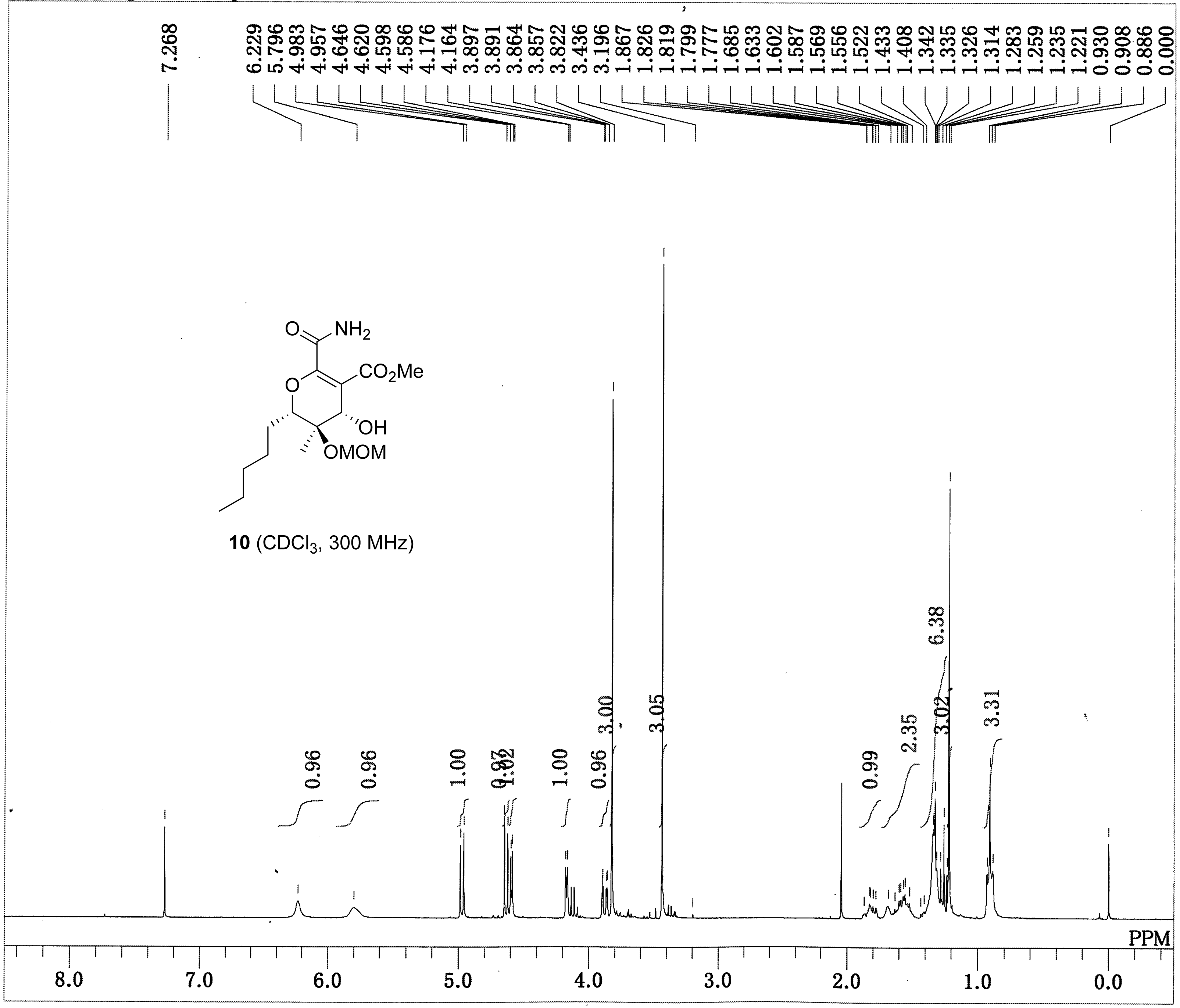

10 ($CDCl_3$, 300 MHz)

DFILE	10-1H.als
COMNT	10-1H
DATIM	Fri Mar 03 14:20:48 2017
OBNUC	1H
EXMOD	NON
OBFRQ	300.40 MHz
OBSET	130.00 KHz
OBFIN	1150.00 Hz
POINT	32768
FREQU	6006.01 Hz
SCANS	16
ACQTM	5.4559 sec
PD	1.5440 sec
PW1	5.60 usec
IRNUC	1H
CTEMP	27.3 c
SLVNT	CDCL3
EXREF	0.00 ppm
BF	0.01 Hz
RGAIN	15

10-13C

C:¥Users¥kogen¥Desktop¥data¥10-13C.als

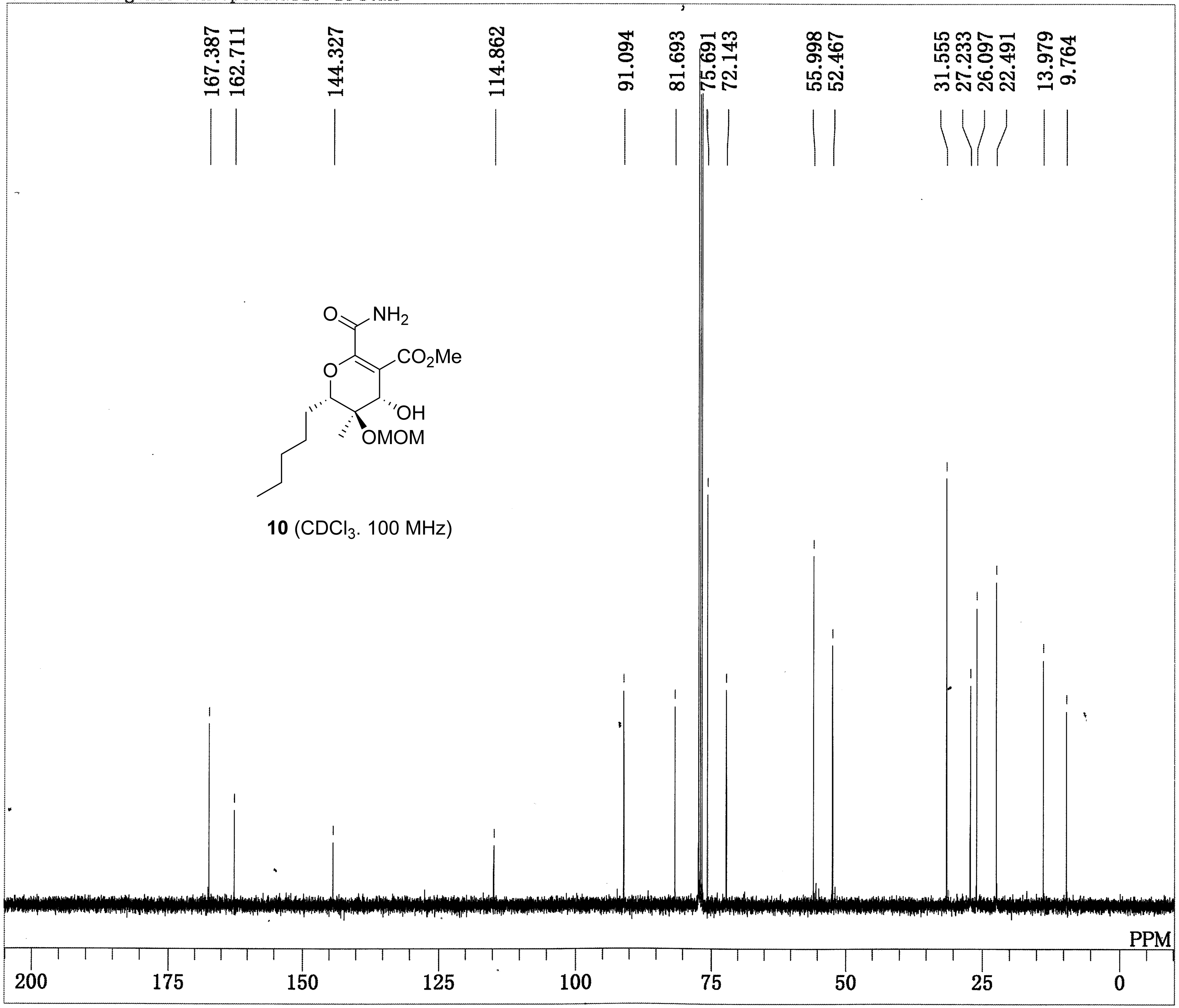

10 ($CDCl_3$. 100 MHz)

DFILE	10-13C.als
COMNT	10-13C
DATIM	Mon Sep 04 19:15:57 2017
OBNUC	13C
EXMOD	BCM
OBFRQ	100.40 MHz
OBSET	125.00 KHz
OBFIN	10500.00 Hz
POINT	32768
FREQU	27118.64 Hz
SCANS	1000
ACQTM	1.2083 sec
PD	1.7920 sec
PW1	5.80 usec
IRNUC	1H
CTEMP	25.5 c
SLVNT	CDCL3
EXREF	77.00 ppm
BF	0.12 Hz
RGAIN	25

11-1H

C:¥Users¥kogen¥Desktop¥data¥11-1H.als

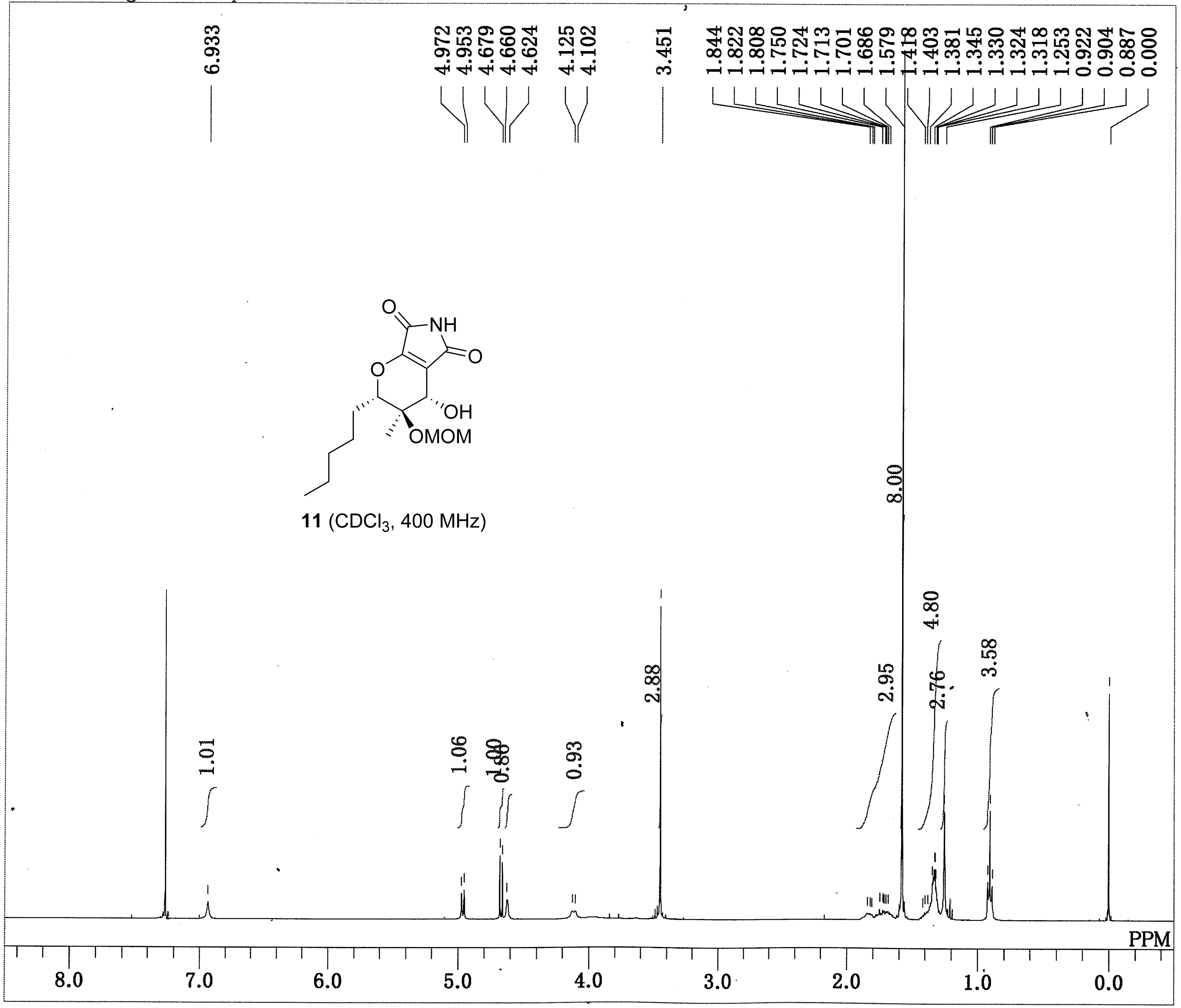

DFILE	11-1H.als
COMNT	11-1H
DATIM	Mon Sep 04 21:28:56 2017
OBNUC	1H
EXMOD	NON
OBFRQ	399.65 MHz
OBSET	124.00 KHz
OBFIN	10500.00 Hz
POINT	16384
FREQU	7992.01 Hz
SCANS	1000
ACQTM	2.0500 sec
PD	4.9500 sec
PW1	5.60 usec
IRNUC	1H
CTEMP	24.5 c
SLVNT	CDCL3
EXREF	0.00 ppm
BF	0.01 Hz
RGAIN	23

11-13C

C:¥Users¥kogen¥Desktop¥data¥11-13C.als

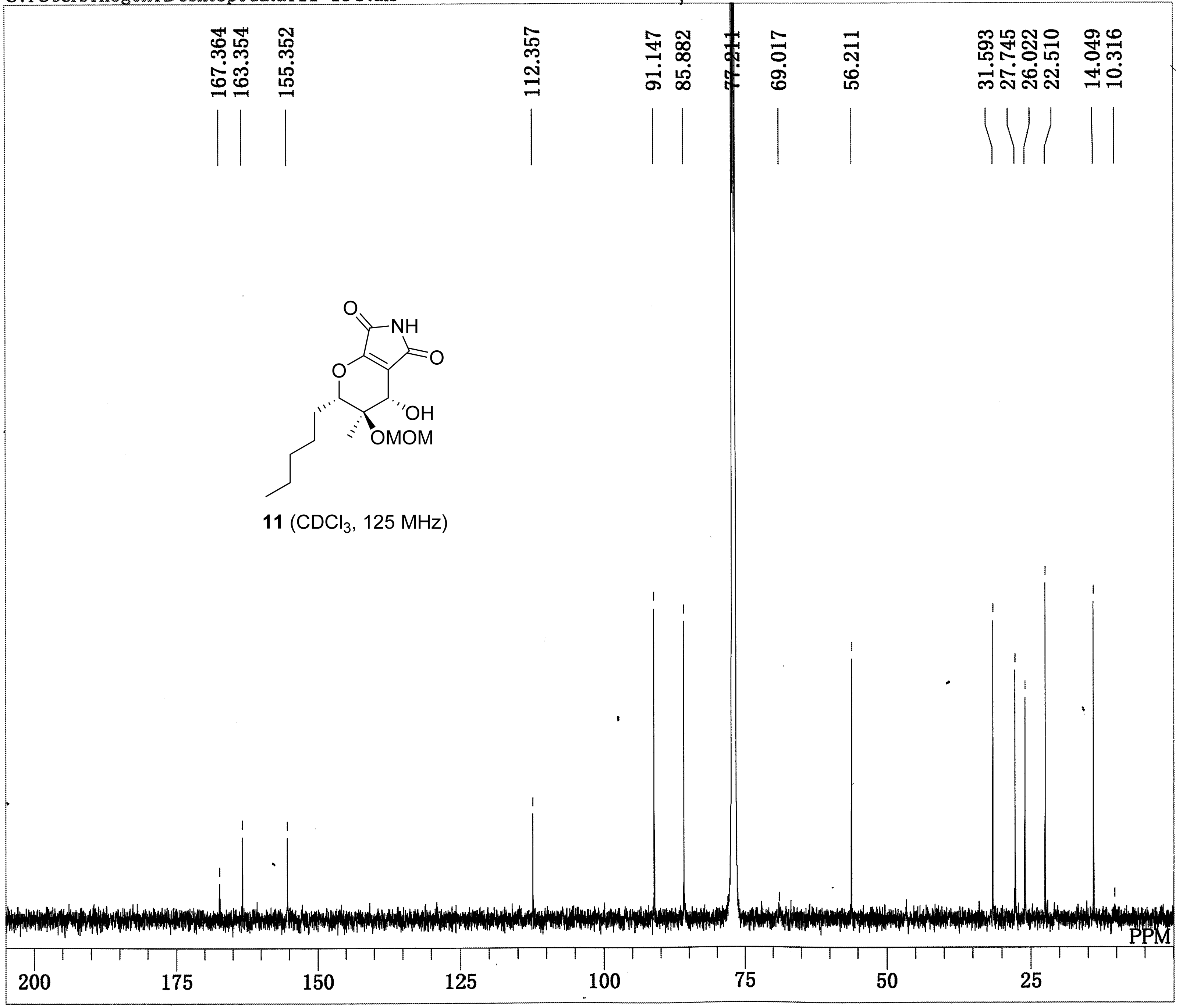

11 ($CDCl_3$, 125 MHz)

DFILE	11-13C.als
COMNT	11-13C
DATIM	2017-09-11 15:17:04
OBNUC	13C
EXMOD	carbon.jxp
OBFRQ	124.51 MHz
OBSET	3.45 KHz
OBFIN	6.00 Hz
POINT	26224
FREQU	31250.00 Hz
SCANS	53969
ACQTM	0.8389 sec
PD	2.0000 sec
PW1	3.42 usec
IRNUC	1H
CTEMP	23.9 c
SLVNT	CDCL3
EXREF	77.00 ppm
BF	1.20 Hz
RGAIN	60

12-1H

C:¥Users¥kogen¥Desktop¥data¥12-1H.als

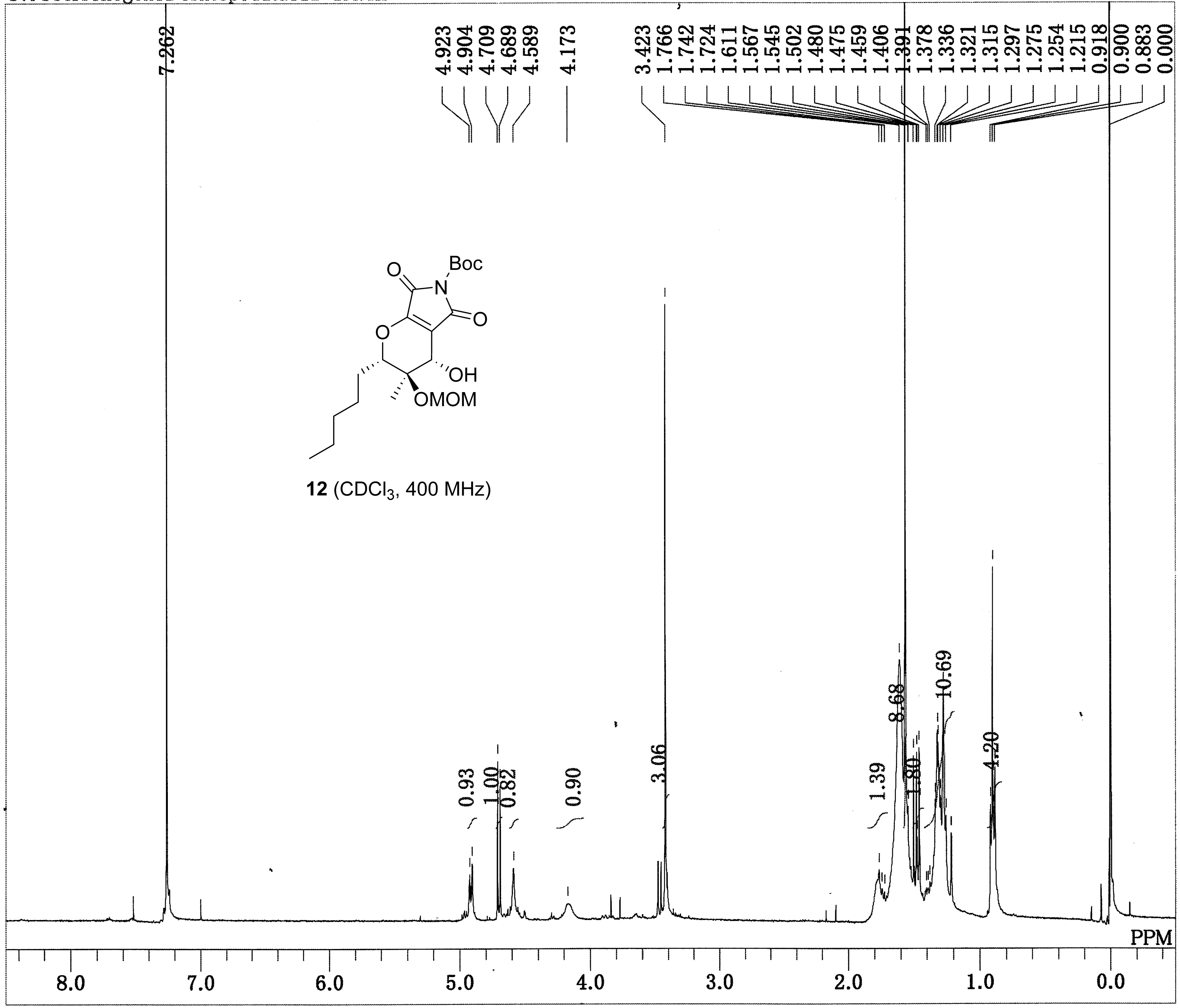

DFILE	12-1H.als
COMNT	12-1H
DATIM	Thu Oct 12 22:35:42 2017
OBNUC	1H
EXMOD	NON
OBFRQ	399.65 MHz
OBSET	124.00 KHz
OBFIN	10500.00 Hz
POINT	16384
FREQU	7992.01 Hz
SCANS	500
ACQTM	2.0500 sec
PD	4.9500 sec
PW1	5.60 usec
IRNUC	1H
CTEMP	23.8 c
SLVNT	CDCL3
EXREF	0.00 ppm
BF	0.12 Hz
RGAIN	24

12-13C

H:¥data¥12-13C.als

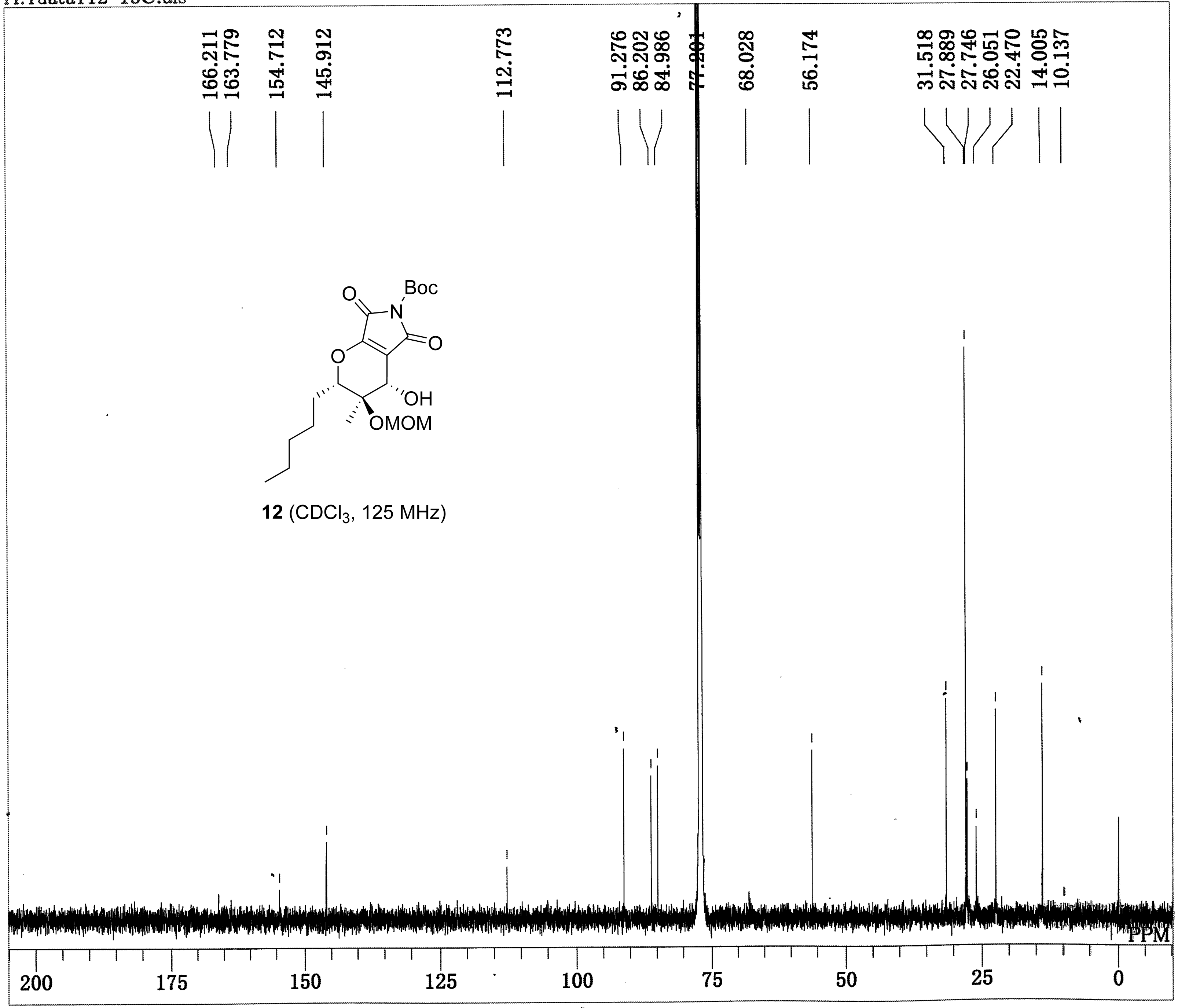

DFILE	12-13C.als
COMNT	12-13C
DATIM	2017-10-25 13:47:47
OBNUC	13C
EXMOD	carbon.jxp
OBFRQ	124.51 MHz
OBSET	3.45 KHz
OBFIN	6.00 Hz
POINT	26214
FREQU	31250.00 Hz
SCANS	58049
ACQTM	0.8389 sec
PD	2.0000 sec
PW1	3.42 usec
IRNUC	1H
CTEMP	24.1 c
SLVNT	CDCL3
EXREF	77.00 ppm
BF	1.12 Hz
RGAIN	60

13-1H

C:¥Users¥kogen¥Desktop¥data¥13-1H.als

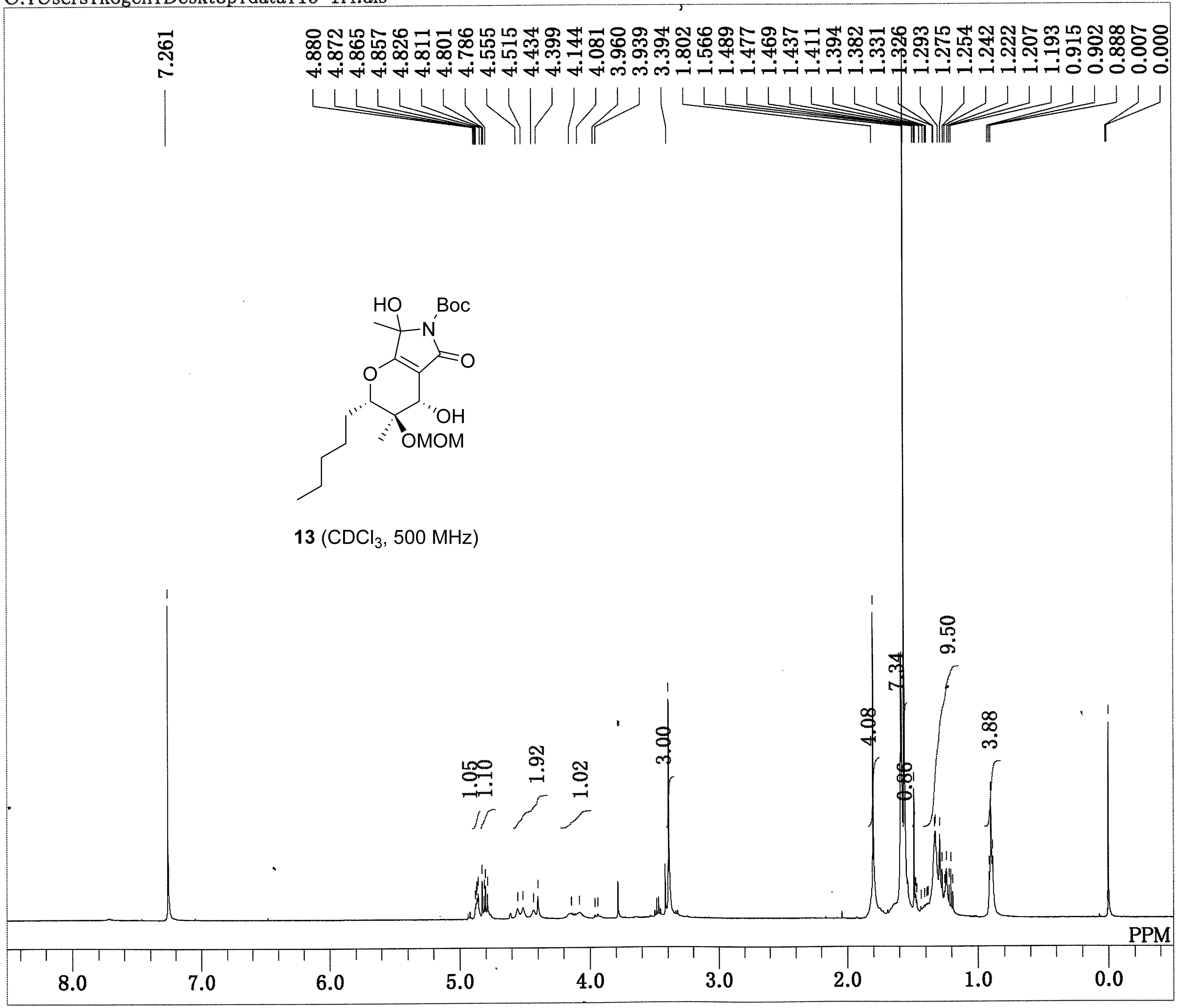

13 ($CDCl_3$, 500 MHz)

DFILE	13-1H.als
COMNT	13-1H
DATIM	Fri Aug 25 07:33:27 2017
OBNUC	1H
EXMOD	non
OBFRQ	500.00 MHz
OBSET	160.00 KHz
OBFIN	2160.00 Hz
POINT	32768
FREQU	10000.00 Hz
SCANS	64
ACQTM	3.2768 sec
PD	3.7232 sec
PW1	5.00 usec
IRNUC	1H
CTEMP	28.4 c
SLVNT	CDCL3
EXREF	0.00 ppm
BF	0.01 Hz
RGAIN	23

13-13C

C:¥Users¥kogen¥Desktop¥data¥13-13C.als

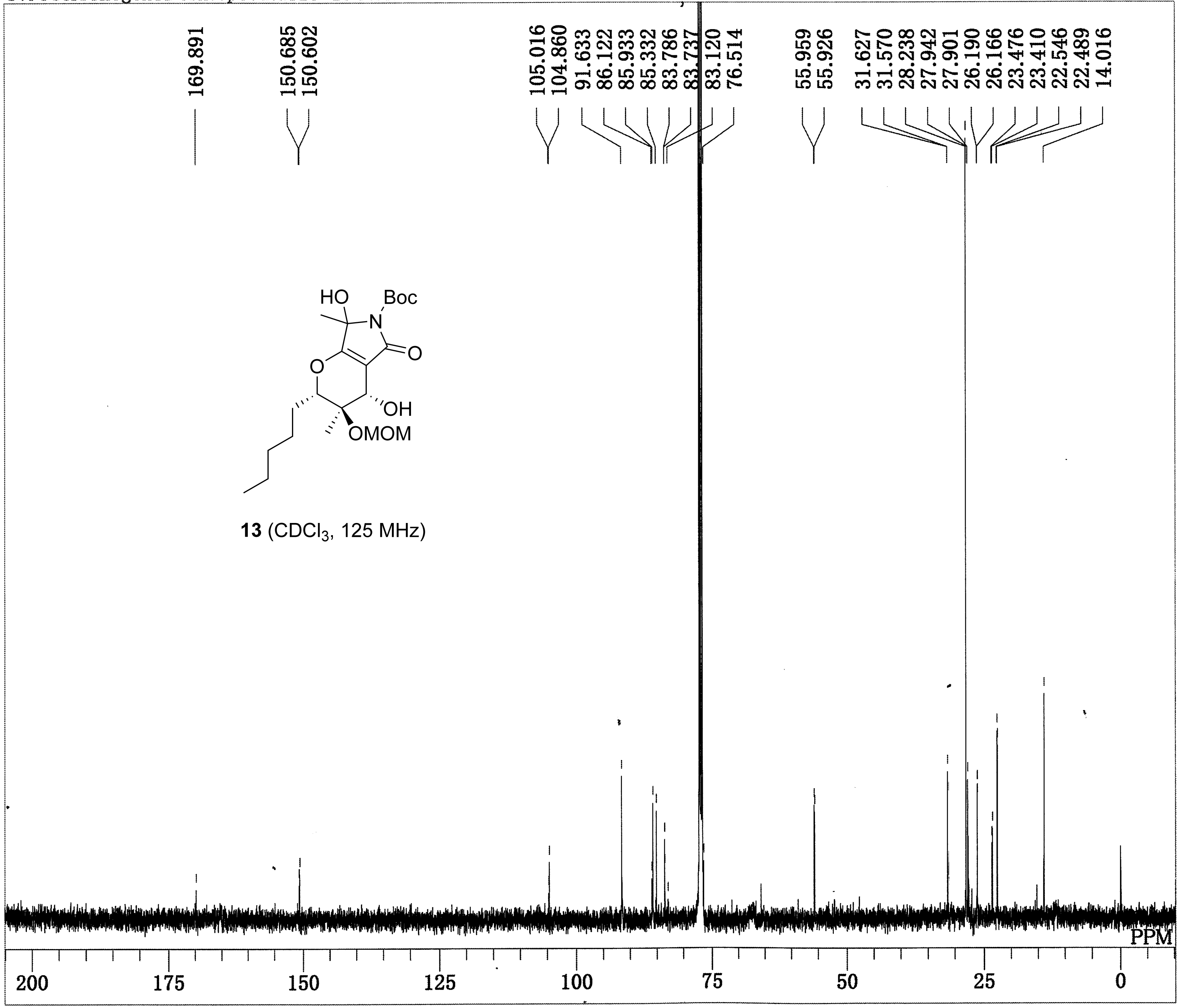

DFILE	13-13C.als
COMNT	13-13C
DATIM	Fri Aug 25 20:54:55 2017
OBNUC	13C
EXMOD	bcm
OBFRQ	125.65 MHz
OBSET	120.00 KHz
OBFIN	7958.00 Hz
POINT	32768
FREQU	33898.30 Hz
SCANS	16000
ACQTM	0.9667 sec
PD	2.0333 sec
PW1	4.40 usec
IRNUC	1H
CTEMP	29.7 c
SLVNT	CDCL3
EXREF	0.00 ppm
BF	0.01 Hz
RGAIN	29

14-1H

C:¥Users¥kogen¥Desktop¥data¥14-1H.als

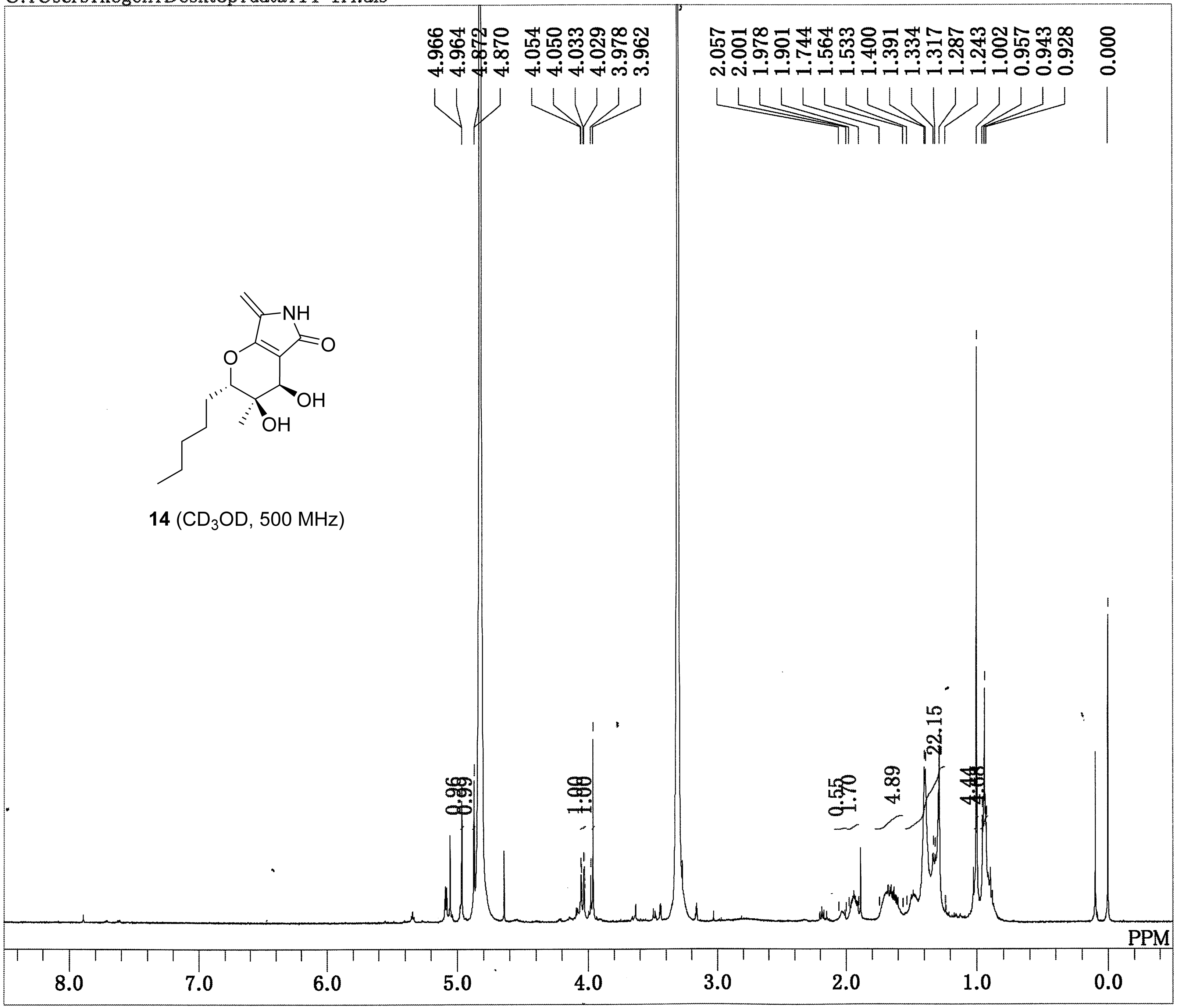

DFILE	14-1H.als
COMNT	14-1H
DATIM	Sun Jan 14 20:05:20 2018
OBNUC	1H
EXMOD	non
OBFRQ	500.00 MHz
OBSET	160.00 KHz
OBFIN	2160.00 Hz
POINT	32768
FREQU	10000.00 Hz
SCANS	256
ACQTM	3.2768 sec
PD	3.7232 sec
PW1	5.00 usec
IRNUC	1H
CTEMP	28.0 c
SLVNT	CD3OD
EXREF	0.00 ppm
BF	0.12 Hz
RGAIN	25

14-13C

C:¥Users¥kogen¥Desktop¥data¥14-13C.als

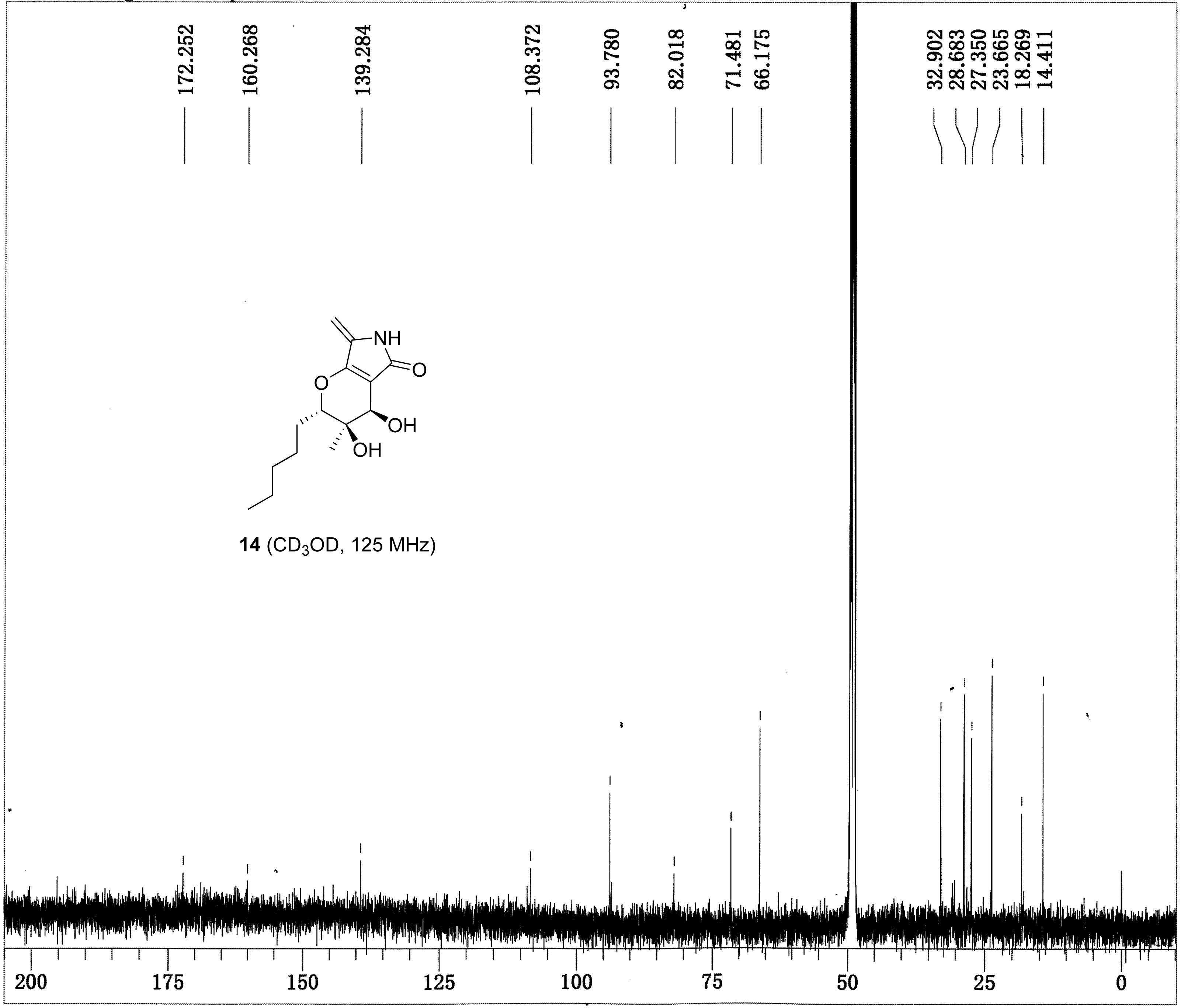

14 (CD_3OD, 125 MHz)

DFILE	14-13C.als
COMNT	14-13C
DATIM	Sun Jan 14 18:27:56 2018
OBNUC	13C
EXMOD	bcm
OBFRQ	125.65 MHz
OBSET	120.00 KHz
OBFIN	7958.00 Hz
POINT	32768
FREQU	33898.30 Hz
SCANS	24000
ACQTM	0.9667 sec
PD	2.0333 sec
PW1	4.90 usec
IRNUC	1H
CTEMP	29.9 c
SLVNT	CD3OD
EXREF	0.00 ppm
BF	1.20 Hz
RGAIN	27

15-1H

C:¥Users¥kogen¥Desktop¥data¥15-1H.als

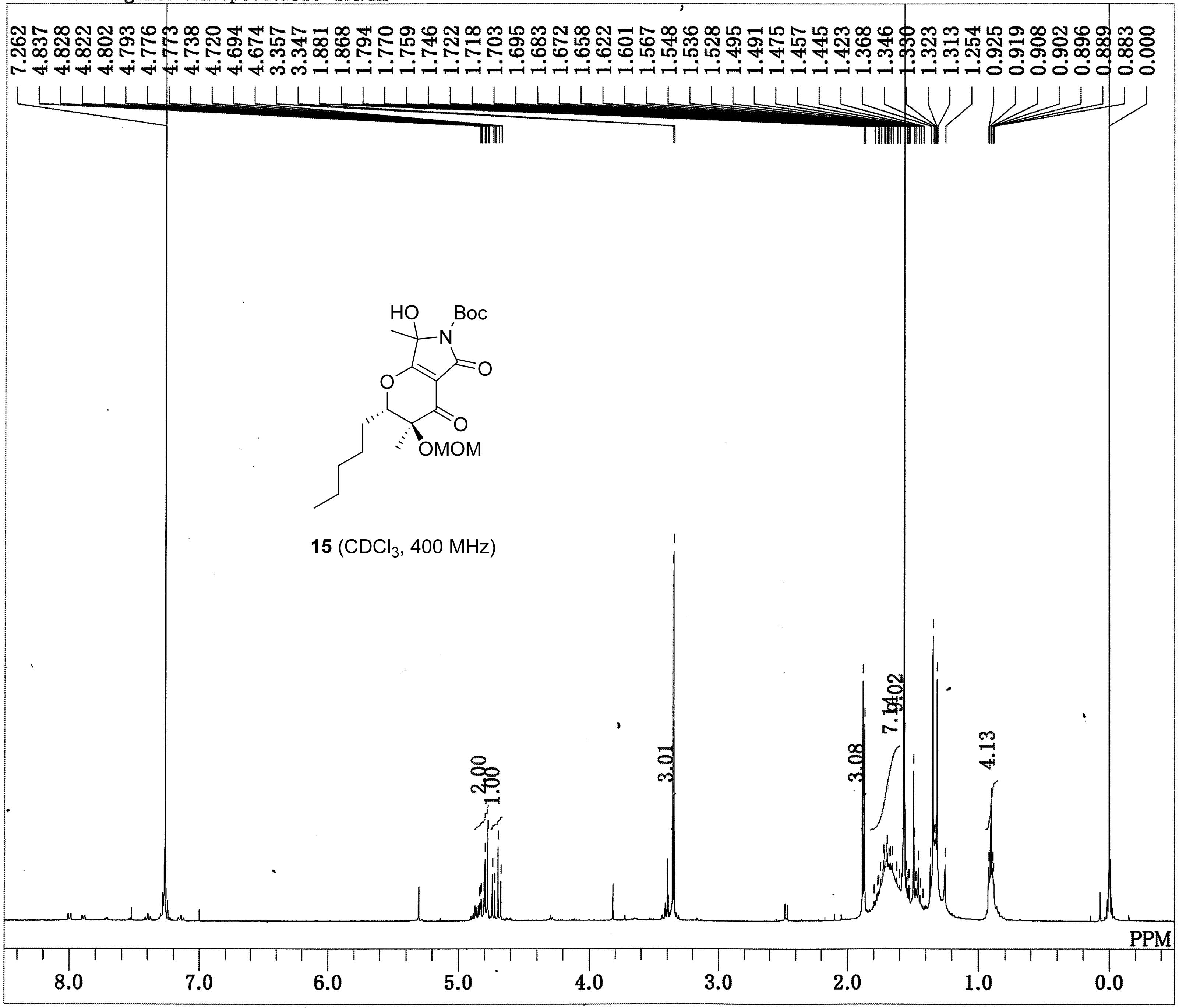

DFILE	15-1H.als
COMNT	15-1H
DATIM	Wed Oct 11 21:34:56 2017
OBNUC	1H
EXMOD	NON
OBFRQ	399.65 MHz
OBSET	124.00 KHz
OBFIN	10500.00 Hz
POINT	16384
FREQU	7992.01 Hz
SCANS	1000
ACQTM	2.0500 sec
PD	4.9500 sec
PW1	5.60 usec
IRNUC	1H
CTEMP	23.4 c
SLVNT	CDCL3
EXREF	0.00 ppm
BF	0.12 Hz
RGAIN	23

15-13C

H:¥data¥15-13C.als

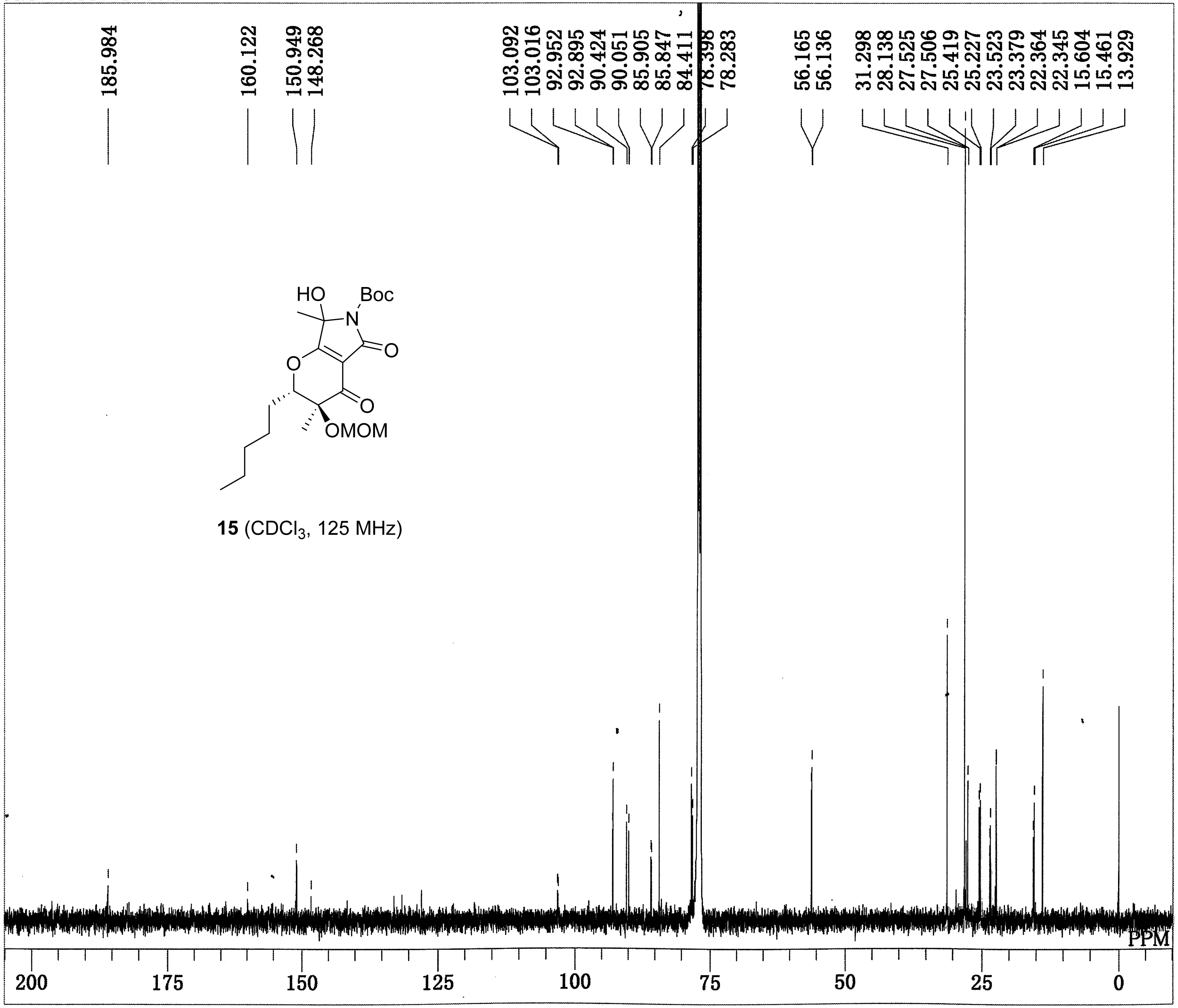

15 ($CDCl_3$, 125 MHz)

DFILE	15-13C.als
COMNT	15-13C
DATIM	2017-10-23 15:57:23
OBNUC	13C
EXMOD	carbon.jxp
OBFRQ	124.51 MHz
OBSET	3.45 KHz
OBFIN	6.00 Hz
POINT	26214
FREQU	31250.00 Hz
SCANS	52693
ACQTM	0.8389 sec
PD	2.0000 sec
PW1	3.42 usec
IRNUC	1H
CTEMP	24.0 c
SLVNT	CDCL3
EXREF	77.00 ppm
BF	1.12 Hz
RGAIN	58

16-1H

C:¥Users¥kogen¥Desktop¥data¥16-1H.als

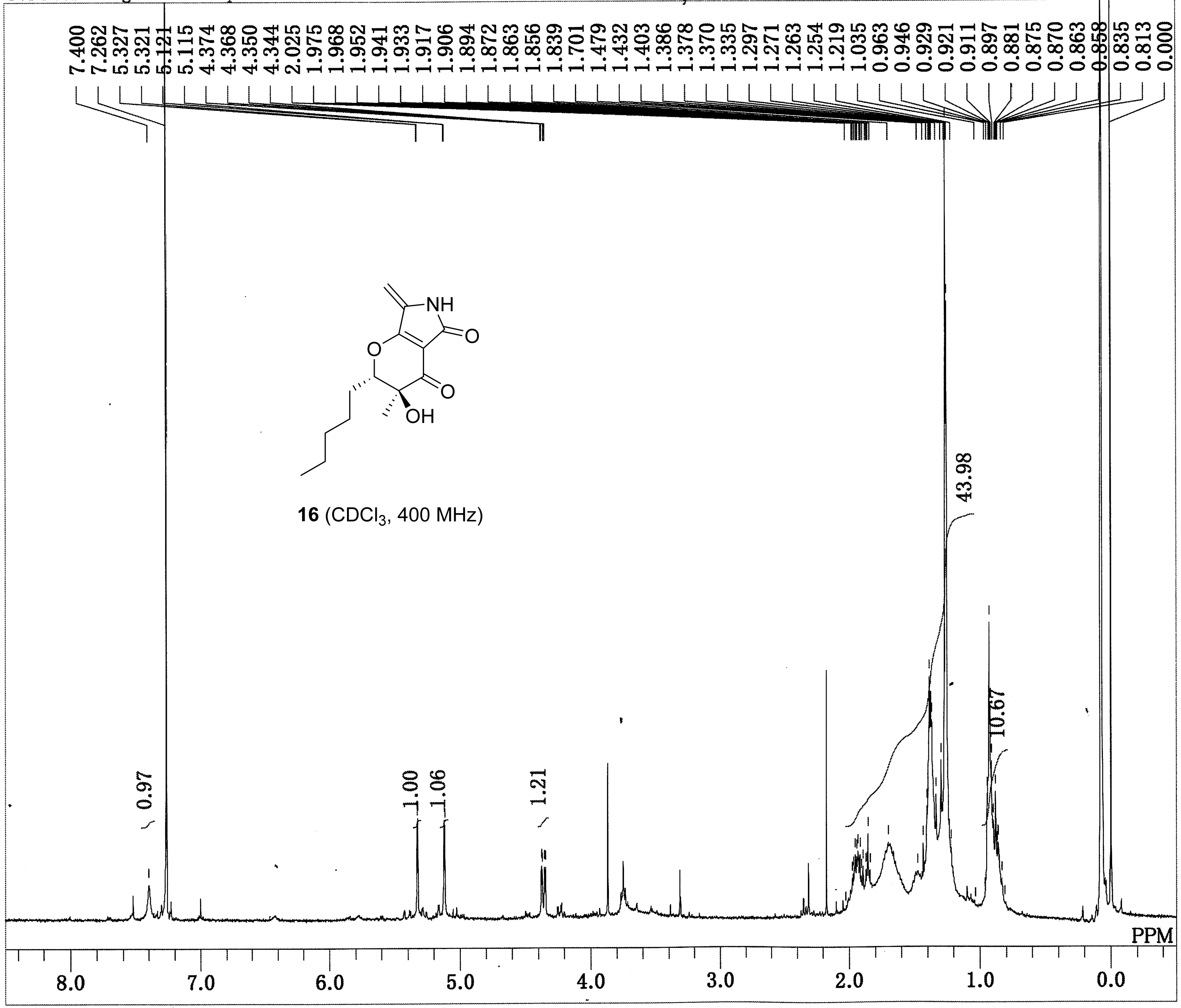

16 ($CDCl_3$, 400 MHz)

DFILE	16-1H.als
COMNT	16-1H
DATIM	Tue Jan 16 14:22:59 2018
OBNUC	1H
EXMOD	NON
OBFRQ	399.65 MHz
OBSET	124.00 KHz
OBFIN	10500.00 Hz
POINT	16384
FREQU	7992.01 Hz
SCANS	100
ACQTM	2.0500 sec
PD	4.9500 sec
PW1	5.80 usec
IRNUC	1H
CTEMP	22.7 c
SLVNT	CDCL3
EXREF	0.00 ppm
BF	0.12 Hz
RGAIN	23

16-13C

C:¥Users¥kogen¥Desktop¥data¥16-13C.als

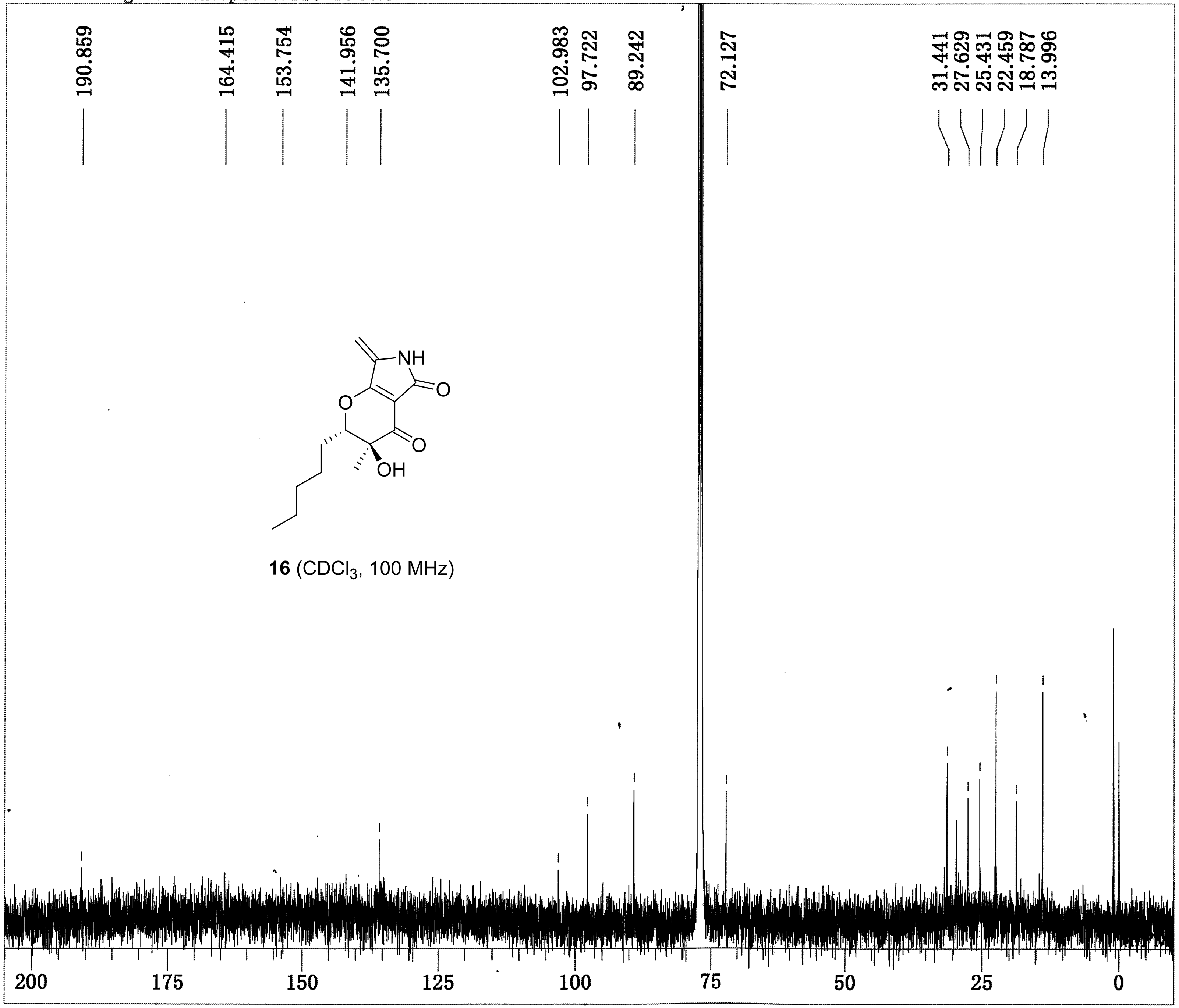

DFILE	16-13C.als
COMNT	16-13C
DATIM	Sat Dec 23 11:27:23 2017
OBNUC	13C
EXMOD	BCM
OBFRQ	100.40 MHz
OBSET	125.00 KHz
OBFIN	10500.00 Hz
POINT	32768
FREQU	27118.64 Hz
SCANS	25000
ACQTM	1.2083 sec
PD	1.7920 sec
PW1	5.80 usec
IRNUC	1H
CTEMP	23.3 c
SLVNT	CDCL3
EXREF	0.00 ppm
BF	1.20 Hz
RGAIN	25

17−1H

C:¥Users¥kogen¥Desktop¥data¥17−1H.als

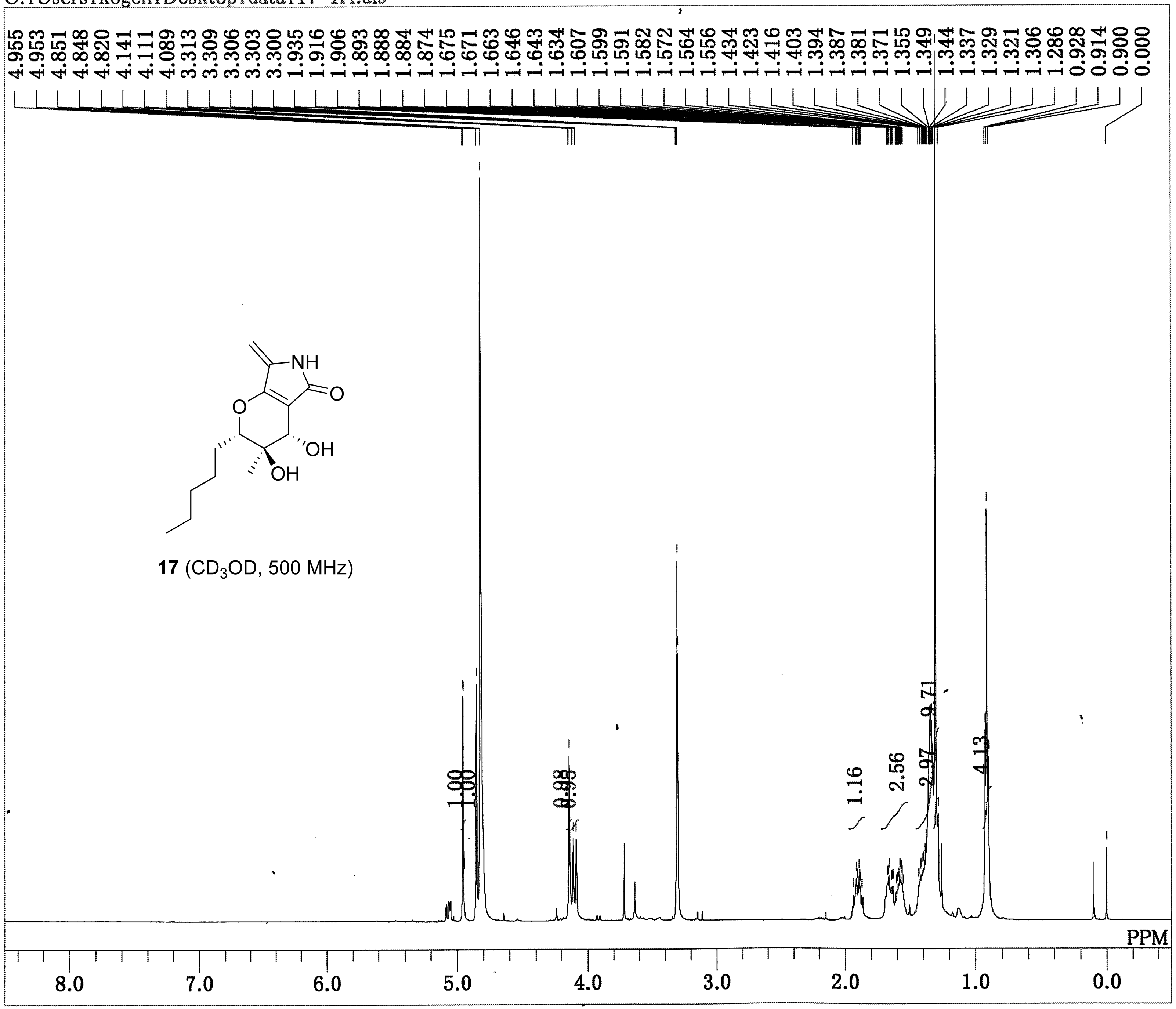

DFILE	17−1H.als
COMNT	17−1H
DATIM	Thu Jan 25 17:05:35 2018
OBNUC	1H
EXMOD	non
OBFRQ	500.00 MHz
OBSET	160.00 KHz
OBFIN	2160.00 Hz
POINT	32768
FREQU	10000.00 Hz
SCANS	64
ACQTM	3.2768 sec
PD	3.7232 sec
PW1	5.00 usec
IRNUC	1H
CTEMP	28.3 c
SLVNT	CD3OD
EXREF	0.00 ppm
BF	0.12 Hz
RGAIN	20

17-13C

C:¥Users¥kogen¥Desktop¥data¥17-13C.als

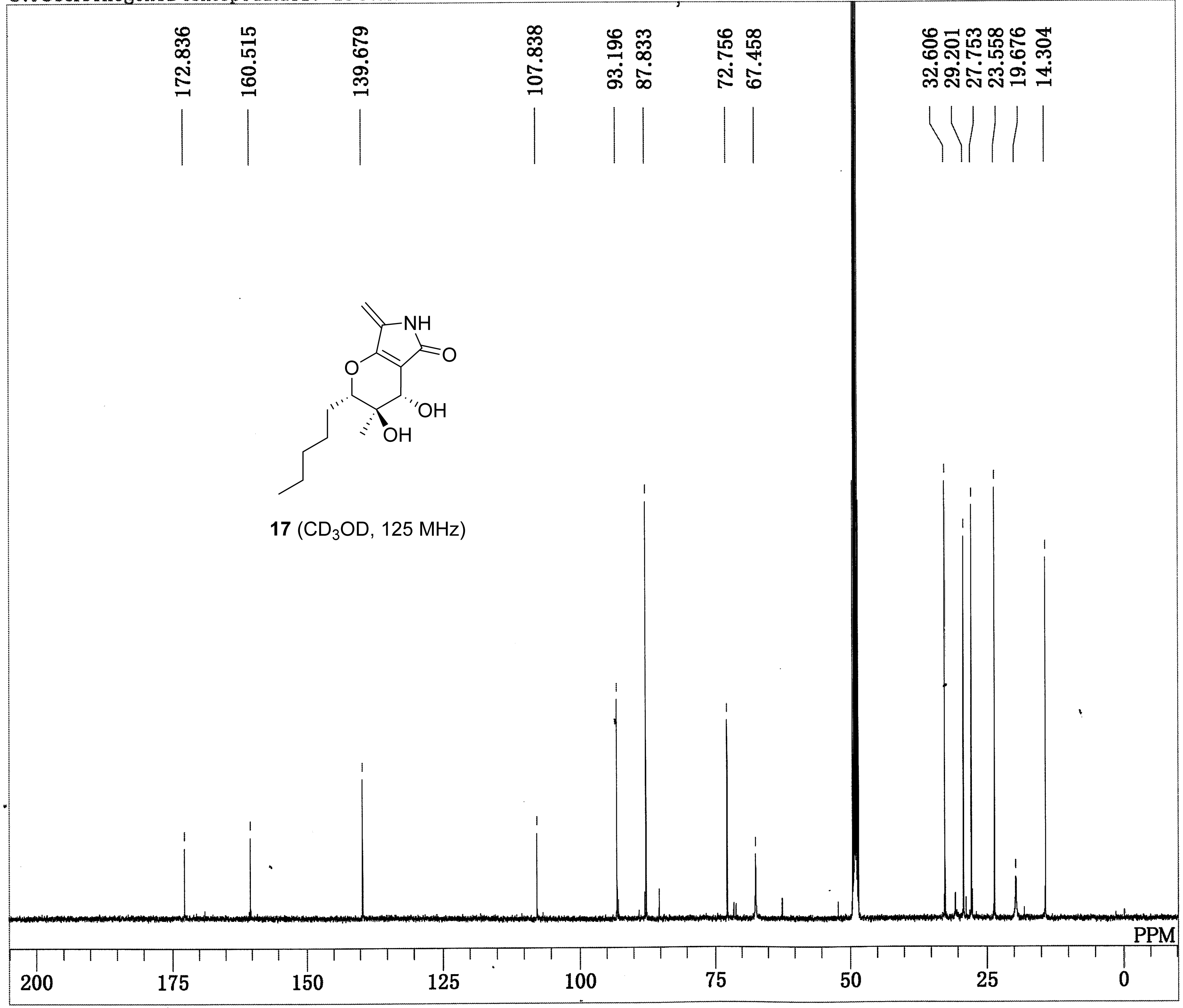

DFILE	17-13C.als
COMNT	17-13C
DATIM	Thu Jan 25 15:47:51 2018
OBNUC	13C
EXMOD	bcm
OBFRQ	125.65 MHz
OBSET	120.00 KHz
OBFIN	7958.00 Hz
POINT	32768
FREQU	33898.30 Hz
SCANS	16000
ACQTM	0.9667 sec
PD	2.0333 sec
PW1	4.90 usec
IRNUC	1H
CTEMP	29.8 c
SLVNT	CD3OD
EXREF	49.00 ppm
BF	0.12 Hz
RGAIN	27

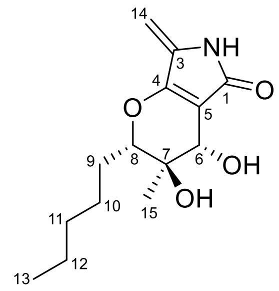

Paraphaeosphaeride C

Position	^{1}H NMR (CD_3OD, 500 MHz)		^{13}C NMR (CD_3OD, 125 MHz)	
	Natural	Synthetic	Natural	Synthetic
1			169.2	172.8
3			140.7	139.7
4			157.2	160.5
5			105.4	107.8
6	4.10 (br s)	4.14 (br s)	66.9	67.5
7			72.8	72.8
8	4.12 (br d, J = 12.0 Hz)	4.10 (br d, J = 11.0 Hz)	87.9	87.8
9a	1.65 (m)	1.66 (m)	29.2	29.2
9b	1.90 (m)	1.90 (m)		
10a	1.42 (m)	1.41 (m)	27.8	27.8
10b	1.56 (m)	1.59 (m)		
11	1.34 (m)	1.34 (m)	32.6	32.6
12	1.34 (m)	1.34 (m)	23.6	23.6
13	0.91 (t, J = 6.0 Hz)	0.91 (t, J = 7.0 Hz)	14.3	14.3
14a	5.04 (br s)	4.94 (d, J = 1.0 Hz)	92.5	93.2
14b	4.89 (br s)	4.85 (d, J = 1.0 Hz)		
15	1.29 (s)	1.31 (s)	19.7	19.7

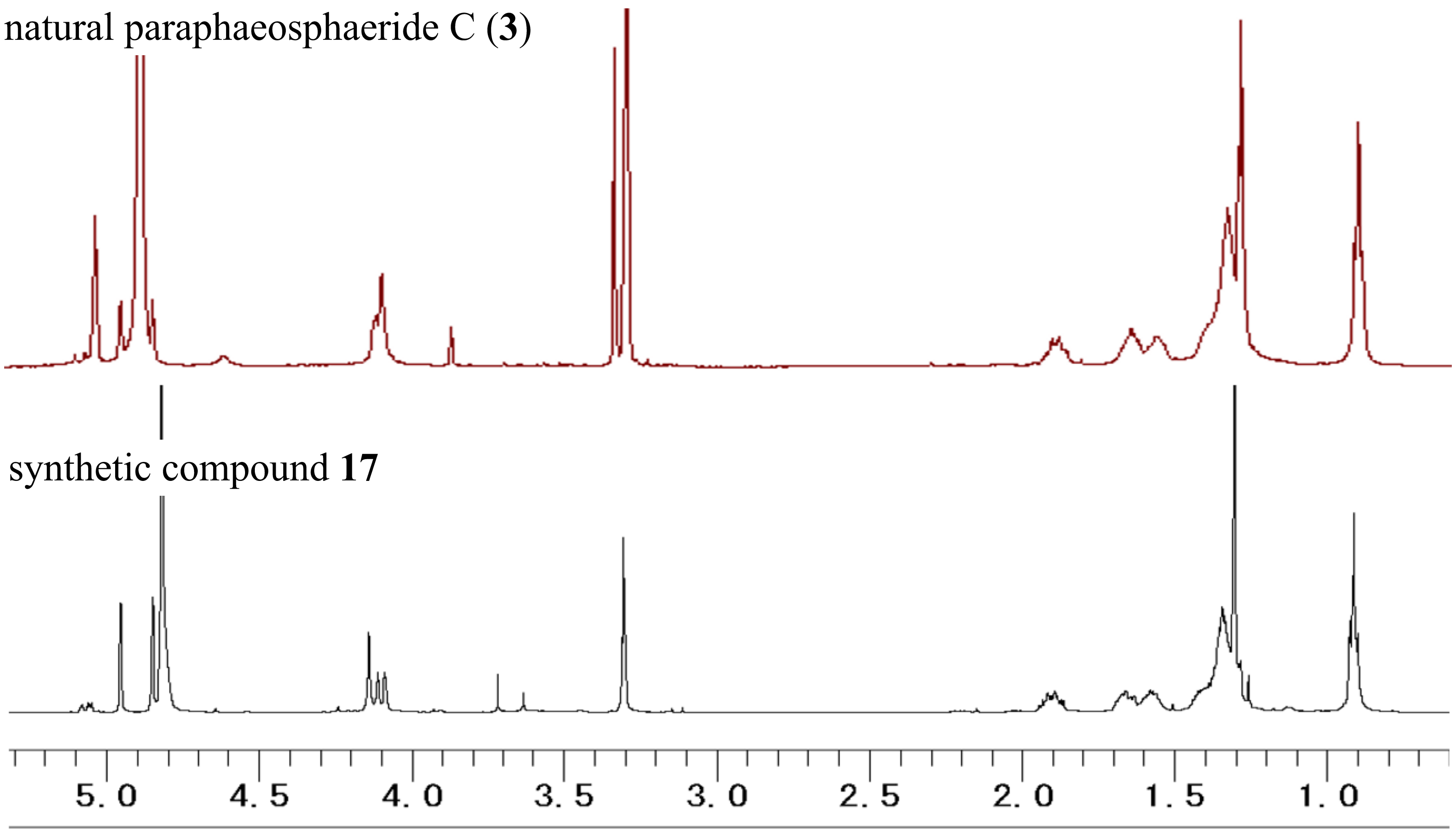

Figure 1. ^{1}H NMR (500 MHz, CD_3OD) comparison of our synthetic compound **17** and natural paraphaeodphaeride C (**3**).

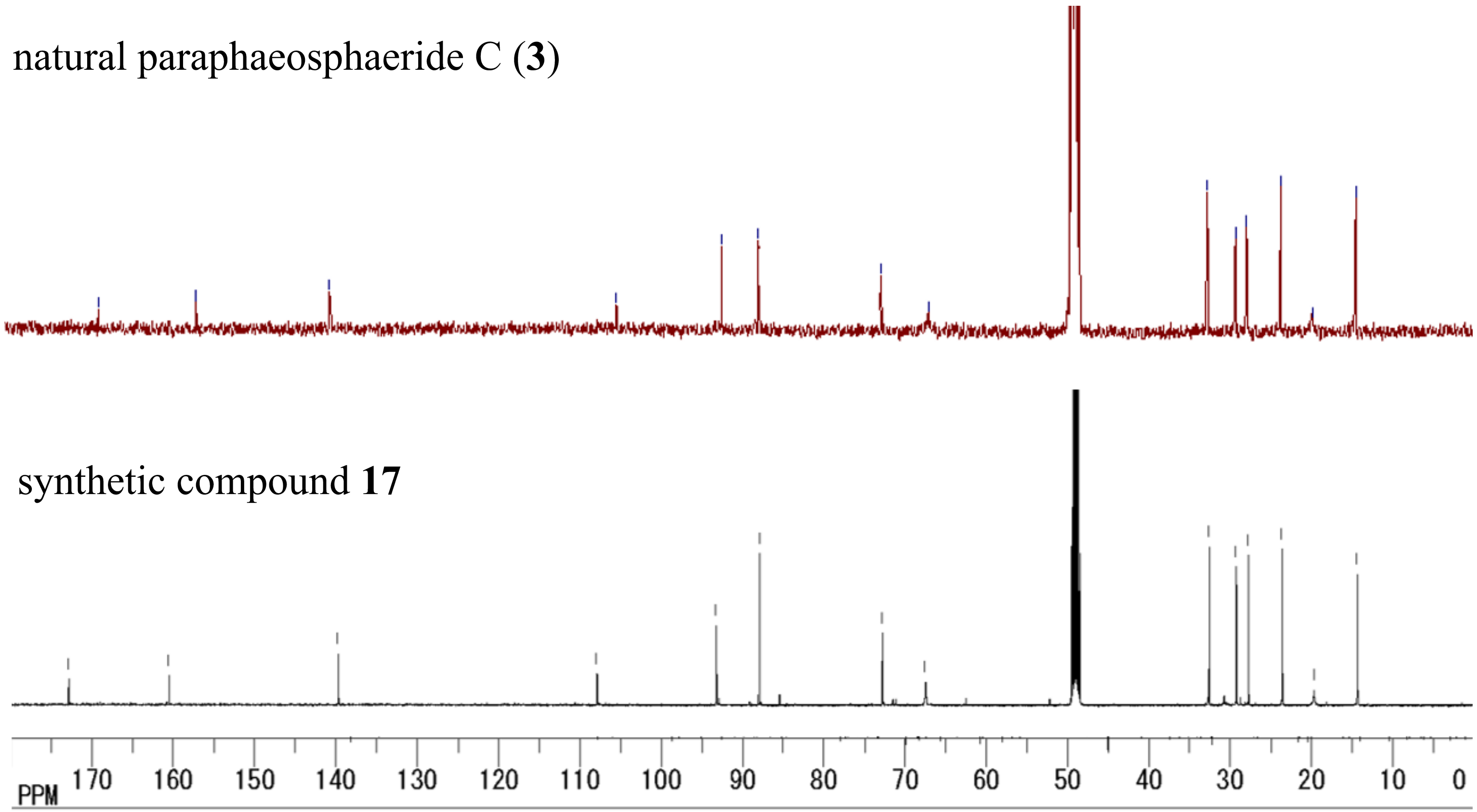

Figure 2. ^{13}C NMR (125 MHz, CD_3OD) comparison of our synthetic compound **17** and natural paraphaeodphaeride C (**3**).
